# Effect of a Culturally Adapted Behavioral Intervention for Latino Adults on Weight Loss Over 2 Years: A Randomized Clinical Trial

**DOI:** 10.1001/jamanetworkopen.2020.27744

**Published:** 2020-12-18

**Authors:** Lisa G. Rosas, Nan Lv, Lan Xiao, Megan A. Lewis, Elizabeth M. J. Venditti, Patricia Zavella, Kristen Azar, Jun Ma

**Affiliations:** 1 Department of Epidemiology and Population Health, Stanford University, Palo Alto, California; 2 Vitoux Program on Aging and Prevention, Department of Medicine, University of Illinois at Chicago, Chicago; 3 Center for Communications Science, RTI International, Seattle, Washington; 4 Department of Psychiatry, University of Pittsburgh School of Medicine, Pittsburgh, Pennsylvania; 5 Department of Latin American and Latino Studies, University of California, Santa Cruz; 6 Sutter Health Center for Health Systems Research, Walnut Creek, California

## Abstract

**Importance:**

Identifying effective weight loss interventions for Latino adults at risk of diabetes is of critical public health importance.

**Objective:**

To determine whether a culturally adapted behavioral intervention for Latino adults was more effective than usual care for weight loss over 24 months.

**Design, Setting, and Participants:**

In this randomized clinical trial, Latino adults with a body mass index (BMI; calculated as weight in kilograms divided by height in meters squared) of 24 or greater and a high risk for type 2 diabetes were recruited in primary care practices in the San Francisco, California, area, randomized to receive the Vida Sana intervention or usual care, and followed up for 24 months. The study was conducted from November 2015 to May 2019, and data were analyzed from July 2019 to Septmeber 2020.

**Interventions:**

The treatment group received Vida Sana, a culturally adapted lifestyle intervention that included a family-based orientation session and 22 group sessions over 12 months. Participants were encouraged to use a wearable activity tracker and mobile applications to track their physical activity and dietary intake. Participants received monthly email messages for an additional 12 months. The control group received usual care.

**Main Outcomes and Measures:**

The primary outcome was weight loss at 24 months. Secondary outcomes included weight loss at 12 months and achieving at least 5% weight loss at 12 and 24 months. Associations of baseline characteristics and intervention adherence with weight loss outcomes were also examined.

**Results:**

Among 191 participants (mean [SD] age, 50.2 [12.2] years; 118 [61.8%] women; 107 participants [57.2%] of Mexican origin; mean [SD] baseline BMI, 32.4 [5.7]) randomized, 92 participants were randomized to the intervention and 99 participants were randomized to usual care. Of these, 185 participants (96.9%) completed 24-month follow-up. Mean (SD) weight loss did not differ significantly by group at 24 months (intervention: −1.1 [5.7] kg; control: −1.1 [7.1] kg; *P* = .93). However, mean (SD) weight loss was significantly greater in the intervention group (−2.6 [6.0] kg) than the control group (−0.3 [4.2] kg) at 12 months (mean difference, −2.1 [95% CI, −3.6 to −0.7] kg; *P* = .005). Intervention participants were more likely to achieve at least 5% weight loss than control participants at 12 months (22 participants [25.9%] vs 9 participants [9.2%]; *P* = .003), and participants who achieved at least 5% weight loss attended more intervention sessions than those who did not (mean [SD], 16.6 [7.6] sessions vs 12.4 [7.5] sessions; *P* = .03).

**Conclusions and Relevance:**

These findings suggest that among Latino adults with high diabetes risk, a culturally adapted behavioral lifestyle intervention was effective for weight loss over 12 months but not 24 months.

**Trial Registration:**

ClinicalTrials.gov Identifier: NCT02459691

## Key Points

**Question:**

Is a culturally adapted behavioral lifestyle intervention using technology more effective than usual care for weight loss among Latino adults in primary care?

**Findings:**

In this randomized clinical trial of 191 Latino patients in primary care, a culturally adapted behavioral lifestyle intervention using technology, including web-based self-monitoring and a wearable activity monitor, was more effective for weight loss over 12 months but not 24 months.

**Meaning:**

These findings suggest that the culturally adapted behavioral intervention using technology was not effective for long-term weight loss over 24 months, so research to optimize intervention effectiveness over 24 months is needed.

## Teaser

This randomized clinical trial examines the effect of a culturally adapted technology-mediated behavioral intervention for Latino adults on weight loss over 2 years.

## Introduction

Latino adults in the United States experience high prevalence of overweight and obesity, with some estimates as high as 80%,^1,2,3^ and disparities in weight-related comorbidities, such as type 2 diabetes, compared with non-Hispanic White adults (12% vs 7%).^4,5,6,7^ Given that Latino people in the US are the largest racial/ethnic minority group and account for the largest share of population growth,^8^ addressing overweight and obesity and related diabetes risk in this group is a critical public health need.^9^

The US Preventive Services Task Force recommends that primary care clinicians refer adults with obesity (body mass index [BMI; calculated as weight in kilograms divided by height in meters squared] ≥30), or those who are overweight (BMI ≥25) and have at least 1 additional cardiovascular disease risk factor, such as prediabetes, to intensive behavioral weight loss interventions.^10^ The Diabetes Prevention Program (DPP) set the criterion standard with an intensive behavioral lifestyle intervention that resulted in 6.8 kg weight loss over 12 months.^11^ Weight loss is the primary driver of reduced diabetes risk.^12^

Latino adults have been underrepresented in studies of weight loss interventions, despite their high risk.^13^ Cultural adaptation of evidence-based interventions, such as the DPP, is important to effectively engage Latino adults owing to cultural differences in factors associated with risk and protection.^14^ A systematic review of culturally adapted DPP interventions for Latino adults documented weight loss at 12 months in 4 studies, ranging from 1.1 kg to 4.2 kg, showing that clinically significant weight loss is possible but of smaller magnitude compared with the original DPP and other studies conducted with primarily non-Hispanic White adults.^15,16,17,18,19,20^ Only 1 study^16^ documented weight loss over 2 years, which was low (1 kg) and not statistically significantly different than weight loss in the control group. The interventions were primarily offered in home or community settings. Primary care settings offer opportunities to leverage clinicians’ relationships with patients to identify and refer those who would benefit from behavioral weight loss interventions.^21^

Technology-mediated weight loss strategies have emerged as an opportunity for improving the effectiveness of weight loss interventions for minority populations.^22,23^ However, studies of culturally adapted behavioral interventions focused on weight loss for Latino adults have not incorporated technology into the interventions. A large portion (80%) of Latino adults report access to the internet, whether by mobile phone, tablet, or computer.^24^ Technology-mediated approaches may increase intervention effectiveness by making the intervention more accessible, but these approaches need to be tested among Latino adults. Thus, the purpose of this randomized clinical trial was to determine whether a culturally adapted behavioral intervention using technology was more effective than usual care for weight loss over 2 years among Latino primary care patients.

## Methods

The institutional review board for Sutter Health, Northern California, approved this study. All participants provided written informed consent. This study is reported following Consolidated Standards of Reporting Trials (CONSORT) reporting guideline for randomized clinical trials.

### Research Design

The Trial Protocol in Supplement 1 and results of the process to culturally adapt the intervention were previously published.^25,26^ Briefly, this was a translational trial in which Latino primary care patients with a BMI of 24 or greater were randomized to receive usual care alone or with a culturally adapted behavioral lifestyle intervention and followed for 24 months with a primary outcome of weight change.

### Study Participants

Participants were recruited from November 2015 to April 2017 in 3 cohorts with 1 each from 3 different primary care sites within the Palo Alto Medical Foundation, a large community-based multispecialty group practice in Northern California. Adult (age ≥18 years) primary care patients who self-reported Latino ethnicity and ability to speak Spanish (Spanish-only or bilingual) with a BMI 24 or greater and prediabetes, a history of gestational diabetes, or 3 of 5 elements of the metabolic syndrome^27^ but without type 1 or type 2 diabetes or cardiovascular disease were eligible to participate. Exclusions included significant psychiatric or medical comorbidities (eg, bipolar disorder, active cancer), pregnancy, or planned relocation during the follow-up period.

### Study Procedures

Active primary care patients who were identified as meeting eligibility criteria in the electronic health record (EHR) received a bilingual (Spanish and English) screening invitation via email or regular mail, if their primary care clinician approved. Patients self-screened online or via telephone with a bilingual study coordinator to assess eligibility criteria. Patients eligible after screening were invited to complete a baseline visit in which height and weight measurements were taken to confirm eligibility. Patients were not recruited on the basis of seeking or being recommended weight management services.

### Randomization and Blinding

Eligible patients were randomized 1:1 to receive usual care or usual care plus a culturally adapted behavioral lifestyle intervention known as *Vida Sana*. All participants were provided with a wearable activity tracker (Fitbit Zip) as an incentive. We used a covariate adaptive block randomization method^28^ for block randomization to achieve balance between treatment groups according baseline clinic site, age, sex, BMI, waist circumference, and level of acculturation assessed by the Short Acculturation Scale for Hispanics.^29^ Investigators, the data and safety monitoring board, outcome assessors, and the data analyst were masked until after completion of the blind review of primary outcome data through 24 months.

### Intervention

The intervention, which was delivered by a trained bilingual health coach in Spanish, is a cultural adaptation of the Group Lifestyle Balance curriculum^30^ derived from the original DPP lifestyle intervention.^11^ A bilingual health coach who did not have a specialized degree was trained by a certified master trainer for the Group Lifestyle Balance curriculum. The in-depth cultural adaptation included focus groups with Latino patients, key informant interviews with clinicians, and a structured pretest with a Latino Patient Advisory Board.^25^ As a result of the adaptation, a family-wide orientation session was instituted to increase awareness about intervention goals and best approaches for providing positive social support, including structural, emotional, appraisal, and informational support. Family members were also included in the session that focused on a healthy home environment to promote desirable food and activity changes.

Intervention sessions were delivered in person for 1 year. Participants used a wearable activity tracker and mobile application to track their physical activity and the MyFitnessPal web or mobile application to track their dietary intake. The first 6 months, or core phase, included 16 group sessions (12 weekly sessions and then 4 bimonthly sessions). Based on social cognitive theory,^31^ the sessions used behavioral strategies, such as self-monitoring, goal setting, stress management, and problem solving, to achieve goals. The goals of the intervention were to achieve 7% weight loss and a minimum of 150 minutes per week of moderate-intensity physical activity. In addition, the health coach provided weekly individualized feedback to participants on their physical activity via their fitness tracker application and diet via their diet tracking application. The postcore support phase included an additional 6 monthly group sessions that focused on continued behavior change and other behavior maintenance strategies (eg, relapse control). A healthy meal was offered at each in-person session to model healthy foods and to increase engagement and retention.^25^ Overall, the first 12 months of the intervention included approximately 23 hours of in-person time with the coach (orientation session plus 22 sessions) as well as feedback via the applications. In the second year, participants were sent monthly emails that reviewed the material from the first year and reminded participants to reach out to the coach for support. All intervention materials were provided in Spanish, with English available on request. Participants could elect to use the smartphone applications in their preferred language.

### Usual Care

Participants in both treatment groups continued to receive usual care from their primary care clinicians. Primary care clinicians were not made aware of patients’ randomization assignment. Clinicians were neither encouraged nor prevented from offering weight management treatment to patients. Participants were not prevented from accessing weight management services from their primary care clinician or in the community. The health care system offered weight management programs, including bariatric surgery, group-based diabetes prevention programs, and meal replacement.

### Outcome Measures

This translational randomized clinical trial minimized patient burden and cost by relying on outcome measures that were easy to measure and required minimal time from participants and staff.^32^ Trained bilingual study coordinators conducted in-person assessments at baseline, 12 months, and 24 months. Assessments were completed in Spanish or English as preferred by the participant. The primary outcome was weight at 24 months, which was measured according to standardized protocols.^33^ Secondary outcomes included weight at 12 months, achieving 5% weight loss at 12 and 24 months, as well as cardiometabolic risk factors, psychosocial well-being, and lifestyle behaviors at 12 and 24 months. Achieving 5% baseline weight loss has been established as clinically meaningful for preventing diabetes.^34^ Cardiometabolic risk factors included blood pressure and waist circumference, measured according to standardized protocols.^35,36,37^ Psychosocial well-being measures included the Obesity-related Problems Scale (range, 0-100),^38,39^ which assesses obesity-specific quality of life, and the 9-item Patient Health Questionnaire (PHQ-9; range, 0-27), which assesses depressive symptoms.^40^ The Obesity-related Problems Scale was translated into Spanish and found to be reliable and valid.^39^ The PHQ-9 has been tested in diverse populations, including Latino adults, and found to be valid.^40^

Lifestyle behaviors included physical activity and diet. Physical activity was assessed using the Stanford 7-Day Physical Activity Recall,^41^ and diet was assessed with a single multiple-pass 24-hour recall using the Nutrition Data System for Research (NDSR).^42,43^ For physical activity, we calculated total minutes and metabolic equivalent task (MET) minutes per week of leisure time physical activity by summing the unweighted and weighted physical activity minutes for moderate (weight: 4 MET minutes), hard (weight: 6 MET minutes), and very hard (weight: 10 MET minutes) activities.^44,45^ We also calculated total energy expenditure, expressed as kilocalories per kilogram per day, by summing MET-minutes per day for each level of physical activity intensity and converting to kilocalories per kilogram per day using the conversion 1 MET-minute = 1 kcal/kg/h.^44,45^ We chose dietary indicators that reflected intervention goals of increasing diet quality and fruit and vegetable consumption and decreasing overall calories and fat. We calculated a Dietary Approach to Stop Hypertension (DASH) score and daily fruit and vegetable servings, daily calories (in kilocalories), and daily fat intake (in grams). All diet variables were derived from the NDSR software. The DASH score was based on 9 nutrient targets (ie, total fat, saturated fat, protein, cholesterol, fiber, magnesium, calcium, sodium, and potassium).^46^ For each nutrient target, participants were assigned a point if they achieved the target and half a point if they achieved an intermediate target (ie, half-way between the DASH target and the population mean) and the DASH score was the sum of points for all 9 nutrients.^47,48^

Participation in outside weight management programs was assessed at study conclusion. Adverse events were monitored at each follow-up point and were also reported ad hoc by interventionists.

### Statistical Analysis

Three categories of prespecified analyses were performed: between-group differences in primary and secondary outcomes, moderation analysis for primary outcome, and session attendance and its association with weight change.

Analyses of between-group differences in primary and secondary outcomes included all participants with follow-up data at 12 or 24 months, and participants were analyzed based on the group to which they were assigned. Tests of group differences at 12 and 24 months in repeated-measures mixed-effects linear models were performed. The fixed effects of each model included baseline value of the outcome, randomization covariates, group (intervention or control), time (12 or 24 months), and group-by-time interaction. In addition, we included Mexican origin as a fixed effect for dietary outcomes, as dietary outcomes can vary among individuals from different countries of origin. The random effects accounted for repeated measures with an unstructured covariance matrix and clustering of patients by primary care clinician. Adjusted differences in mean changes with 95% CIs and Cohen *d* for the primary outcome were obtained using model-based estimates. Analyses used all available data for each outcome, and missing data were handled directly through maximum-likelihood estimation via mixed modeling. Per the Trial Protocol in Supplement 1, in the case of missing study-measured weight, the closest EHR weight within 3 months of the due date of a missed study visit or self-reported weight (if no EHR weight available) was used.^49^ Sensitivity analyses were conducted using study-measured weights only. A bootstrap resampling method was used to verify that mixed-model–based results were not sensitive to violations of modeling assumptions.^50^ Because of the potential for type 1 error due to multiple comparisons, findings for analyses of secondary outcomes should be interpreted as exploratory.

Moderation analysis used the same mixed-effects linear model, as well as the main effect of each potential effect modifier and its interaction with group; the latter, if significant, resulted in a rejection of the null hypothesis of no moderation. We then added the 3-way interaction of time, group, and moderator to generate time-specific moderation effects. Potential effect modifiers included sociodemographic factors (ie, age, sex, education, employment, occupation, marital status, household size, and income), food security (ie, household food security scale, which defined high food security as a yes or no category), acculturation (assessed with the Short Acculturation scale for Hispanics^29^), and health literacy (assessed with a short assessment of health literacy in Spanish and English), all of which were prespecified.^26^

Among participants randomized to the intervention, we examined 4 indicators of intervention adherence and engagement and their correlation with weight loss: group session attendance, monitoring of weight at sessions, self-monitoring of diet in the diet tracking application, and self-monitoring of physical activity in the wearable activity tracker application. For group session attendance, the correlation between the total number of attended group sessions and weight changes from baseline to 12 and 24 months for the intervention group were plotted with the fitted regression lines. Monitoring of weight was determined based on whether the patient weighed in at the session. Self-monitoring of diet and physical activity were determined based on whether the coach recorded the patient’s self-monitoring data at the session. We categorized patients into 3 groups of adherence based on if they had data in less than 50% of the sessions, 50% to less than 75% of the sessions, or 75% or more of the sessions. We compared the mean weight changes from baseline to 12 and 24 months in the 2 highest groups of adherence to the lowest using *t* tests.

As estimated, 93 participants would be needed per group to provide 80% power to detect a net between-group mean (SD) difference of 2.1 (4.6) kg at 24 months, assuming α = 5% (2-sided) and 80% retention based on a previous trial in the same health care system.^51^ All analyses were conducted using SAS Enterprise Guide version 7.1 (SAS Institute). Statistical significance was defined by 2-sided *P* < .05. Data were analyzed from July 2019 to September 2020.

## Results

### Study Participants

**Figure 1.**
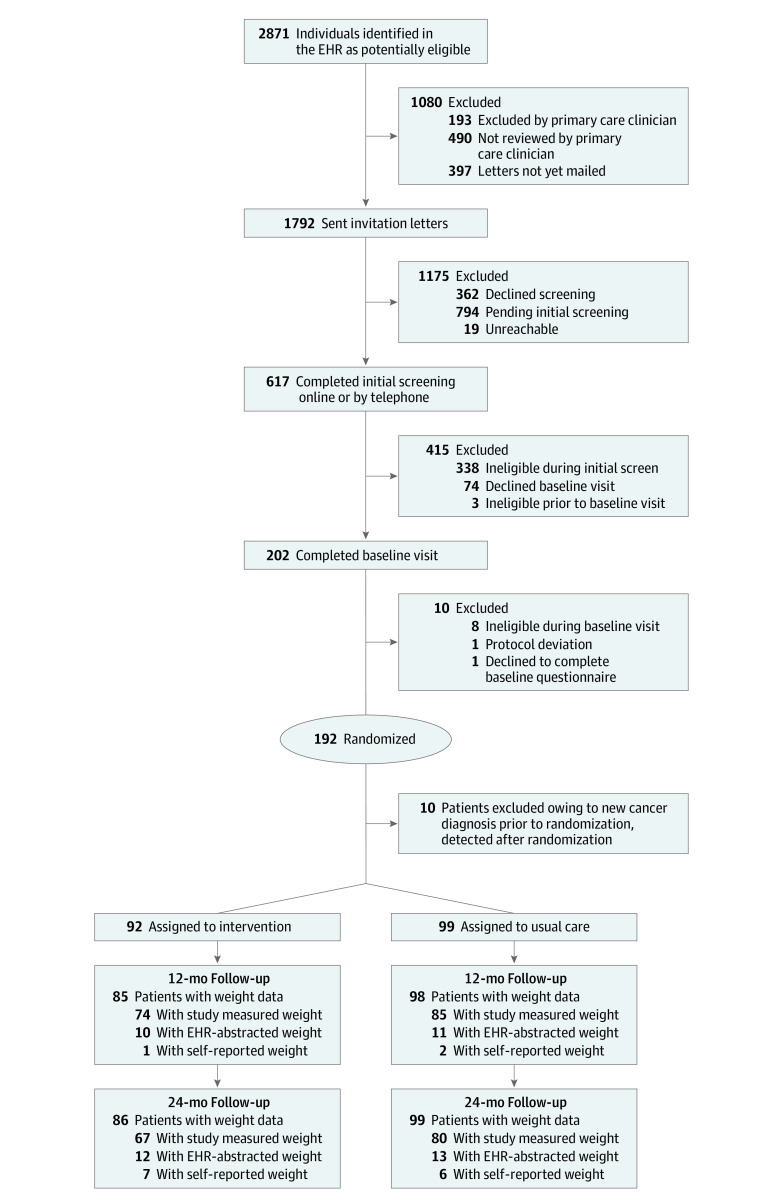
Participant Enrollment Flowchart EHR indicates electronic health record.

A total of 192 participants were randomized. After excluding one participant postrandomization owing to a new cancer diagnosis that occurred before the patient was randomized but was not reported to the study until afterwards, 191 participants (mean [SD] age, 50.2 [12.2] years; 118 [61.8%] women; 107 participants [57.2%] of Mexican origin) were included, with 92 participants randomized to receive the intervention and 99 participants randomized to receive usual care (Table 1). Primary outcome analyses included participants with follow-up data at 12 or 24 months, including 91 participants in the intervention group and 99 participants in the usual care group (Figure 1).

**Table 1.** Demographic and Key Baseline Characteristics by Treatment Group^a^

Characteristic	Mean (SD)		
	Overall (n = 191)	Intervention (n = 92)	Usual care (n = 99)
Age, y	50.2 (12.2)	50.3 (12.5)	50.1 (12.0)
Female, No. (%)	118 (61.8)	57 (62)	61 (61.6)
Region of origin, No. (%)^b^			
Mexico	107 (57.2)	52 (57.1)	55 (57.3)
Central America	32 (17.1)	17 (18.7)	15 (15.6)
South America	22 (11.8)	8 (8.8)	14 (14.6)
Puerto Rico, Cuba, other Spanish, or multi-origin	26 (13.9)	14 (15.4)	12 (12.5)
Education, No. (%)^c^			
≤High school or GED	54 (28.9)	26 (28.9)	28 (28.9)
Some college	50 (26.7)	22 (24.4)	28 (28.9)
College graduate	41 (21.9)	21 (23.3)	20 (20.6)
Post college	42 (22.5)	21 (23.3)	21 (21.7)
Marital status, No. (%)^d^			
Married or living with a partner	126 (67)	58 (63.7)	68 (70.1)
Single, separated, divorced, or widowed	62 (33)	33 (36.3)	29 (29.9)
Employment status, No. (%)^d^			
Full-time	134 (71.3)	63 (69.2)	71 (73.2)
Part-time	20 (10.6)	15 (16.5)	5 (5.2)
Unemployed	34 (18.1)	13 (14.3)	21 (21.6)
Occupation industry, No. (%)^e^			
Professional and business services	28 (18.8)	13 (16.7)	15 (21.1)
Educational and health services	43 (28.9)	20 (25.6)	23 (32.4)
Information, retail, transportation, construction, manufacturing, hospitality, or public administration	43 (28.8)	25 (32.1)	18 (25.4)
Other	35 (23.5)	20 (25.6)	15 (21.11)
Household size, No. (%)^f^			
<2	11 (5.9)	7 (7.7)	4 (4.2)
2	28 (15.1)	7 (7.7)	21 (22.1)
≥3	147 (79)	77 (84.6)	70 (73.7)
Annual family income, $^g^			
<75 000	73 (44.8)	39 (48.1)	34 (41.5)
75 000 to <125 000	40 (24.5)	18 (22.2)	22 (26.8)
≥125 000	50 (30.7)	24 (29.6)	26 (31.7)
Household food security, No. (%)^d^^,^^h^			
High	158 (84)	80 (87.9)	78 (80.4)
Low	21 (11.2)	7 (7.7)	14 (14.4)
Very low	9 (4.8)	4 (4.4)	5 (5.2)
BMI^a^	32.4 (5.7)	32.4 (5.4)	32.4 (6.0)
Women	33.0 (6.1)	33.3 (6.0)	32.8 (6.1)
Men	31.4 (5.0)	30.8 (3.8)	31.9 (5.9)
Weight, kg	87.1 (19.1)	86.6 (17.2)	87.6 (20.8)
Women	82.3 (17.0)	83.2 (17.4)	81.5 (16.7)
Men	94.8 (19.9)	92.2 (15.5)	97.3 (23.2)
Waist circumference, cm	103.9 (14.4)	103.8 (13.7)	103.9 (15.0)
Women	103.8 (14.5)	104.3 (15.4)	103.5 (13.7)
Men	103.9 (14.3)	103.1 (10.6)	104.7 (17.1)
Short Acculturation Scale for Hispanics score^i^	3.1 (0.8)	3.1 (0.7)	3.1 (0.8)
Short assessment of health literacy score^d^^,^^j^	16.6 (1.8)	16.6 (1.9)	16.6 (1.8)
Blood pressure, mm Hg			
Systolic	121.8 (14.6)	122.5 (12.8)	121.3 (16.2)
Diastolic	74.1 (9.5)	74.8 (8.6)	73.5 (10.2)
Leisure time moderate and vigorous physical activity total min/wk^k^	232 (241)	208 (191)	254 (280)
Leisure time physical activity, MET min/wk^k^^,^^l^	1126 (1249)	1000 (965)	1246 (1463)
Total energy expenditure, kcal/kg/d^k^^,^^m^	35.4 (4.2)	35.4 (4.2)	35.4 (4.1)
DASH score^n^^,^^o^	2.5 (1.3)	2.4 (1.2)	2.6 (1.4)
Fruit and vegetable, servings/d^n^	4.3 (3.7)	3.6 (2.5)	5.0 (4.4)
Total calorie intake, kcal/d^n^	1734 (808)	1623 (657)	1839 (919)
Total fat, g/d^n^	71.4 (41.8)	69.7 (36.9)	73.1 (46.1)
Obesity-related problem scale^d^^,^^p^	40.2 (30.0)	44.0 (28.5)	36.8 (31.1)
PHQ-9 score ^d^^,^^q^	5.4 (4.5)	6.0 (4.7)	4.8 (4.3)
T-score^d^^,^^r^			
Sleep disturbance	51.6 (8.9)	51.6 (9.3)	51.5 (8.6)
Sleep impairment	51.2 (9.2)	51.6 (9.3)	50.7 (9.1)

Abbreviations: BMI, body mass index (calculated as weight in kilograms divided by height in meters squared); DASH, Dietary Approach to Stop Hypertension; GED, general educational development; MET, metabolic equivalent task; PHQ-9, Patient Health Questionnaire 9-item.

^a^ Prognostic factors for randomization were study site, age, sex, BMI, waist circumference, and Short Acculturation Scale for Hispanics.

^b^ Includes data for 91 individuals in the intervention group and 96 individuals in the usual care group.

^c^ Includes data for 90 individuals in the intervention group and 97 individuals in the usual care group.

^d^ Includes data for 91 individuals in the intervention group and 97 individuals in the usual care group.

^e^ Includes data for 71 individuals in the intervention group and 78 individuals in the usual care group.

^f^ Includes data for 91 individuals in the intervention group and 95 individuals in the usual care group.

^g^ Includes data for 81 individuals in the intervention group and 82 individuals in the usual care group.

^h^ Scores were calculated as the sum of affirmative responses to 6 questions in the module, then food security status was assigned as raw score 0 to 1, high or marginal food security (raw score 1 may be considered marginal food security, but a large proportion of households that would be measured as having marginal food security using the household or adult scale will have a raw score of 0 on the 6-item scale); raw score 2 to 4, low food security; and raw score 5 to 6, very low food security.

^i^ Measured using a 12-item questionnaire scaled from 1 to 5 for each item (1 = only Spanish, 2 = Spanish better than English, 3 = both equally, 4 = English better than Spanish, and 5 = only English), and the score is calculated as mean of all items. A higher score indicates higher acculturation to US society.

^j^ 18 test items to assess a Spanish-speaking adult’s ability to read and understand common medical terms in English or an English-speaking adult’s ability to read and understand common medical terms in Spanish. Each correct answer gets 1 point. The score is the sum of all items, ranging from 0 to 18.

^k^ Includes data for 92 individuals in the intervention group and 97 individuals in the usual care group.

^l^ Physical activity levels were measured by the interview-administered Stanford 7-Day Physical Activity Recall. Leisure-time physical activity = non–work-related moderate activity min/wk × 4 METs + hard activity min/wk × 6 METs + very hard activity min/wk × 10 METs. One MET is defined as the energy expenditure for sitting quietly.

^m^ The Stanford 7-Day Physical Activity Recall data also provided estimates of total daily energy expenditures. Total energy expenditure = sleep hours × 1 MET + light activity hours × 1.5 METs + moderate activity hours × 4 METs + hard activity hours × 6 METs + very hard activity hours × 10 METs.

^n^ Includes data for 76 individuals in the intervention group and 81 individuals in the usual care group.

^o^ DASH scores were calculated based on combining 9 nutrient targets (ie, total fat, saturated fat, protein, cholesterol, fiber, magnesium, calcium, sodium, and potassium). The intermediate target of each nutrient was halfway between the DASH target and population mean (based on the National Health and Nutrition Examination Surveys 2007-2008, latest data available at the inception of this study). For a nutrient, participants reaching the DASH target were assigned 1 point, those reaching the intermediate target were assigned a half point, and those not meeting the intermediate target were given 0 points. The DASH score was the sum of points for all 9 nutrients.

^p^ Calculated as mean scores of 8 questions, with a range from 0 (no at all) to 3 (extremely). Raw scores were multiplied by 100 then divided by 3. Higher score indicates more obesity-related psychosocial problems.

^q^ Calculated as total scores of the 9 items with a range from 0 (no symptoms) to 27 (most severe symptoms). PHQ-9 score of 0 to 4 indicates minimal depression; 5 to 9, mild depression; 10 to 14, moderate depression; 15 to 19, moderately severe depression; and 20 to 27, severe depression.

^r^ Measured using a population scale with a range from 25 (extreme disturbance or impairment) to 80 (none) converted from the raw total score of 8-item questionnaire. A score of 50 represents the mean of the calibration sample, which was generally more enriched for chronic illness.

Baseline demographic and clinical characteristics were similar between participants randomized to the intervention and usual care, except the intervention group, compared with the usual care group, had fewer unemployed adults (13 participants [14.3%] vs 21 participants [21.6%]), smaller household size (<2 members/household: 7 participants [7.7%] vs 4 participants [4.2%]), and less consumption of fruits and vegetables per day (mean [SD], 3.6 [2.5] servings/d vs 5.0 [4.4] servings/d). Most participants had at least some college experience (133 participants [71.1%]) and middle to upper incomes (90 participants [55.2%] with annual family incomes >$75 000) (Table 1). Participants’ mean (SD) BMI was 32.4 (5.7) overall and slightly higher among women compared with men (33.0 [6.1] vs 31.4 [5.0]).

### Primary and Secondary Outcomes

There was only 1 (0.5%) missing weight measurement. Mean (SD) weight loss at 24 months did not differ significantly among intervention participants compared with control participants (−1.1 [7.1] kg vs −1.1 [5.7] kg; adjusted mean difference, 0.1 [95% CI, −1.8 to 1.9] kg; *P* = .93) (Table 2). Sensitivity analyses using only study-measured weights and bootstrap resampling method provided consistent results (eTable 1 and eTable 2 in Supplement 2). However, mean (SD) weight loss at 12 months was significantly greater among intervention participants compared with control participants (−2.6 [6.0] kg vs−0.3 [4.2] kg; adjusted mean difference, −2.1 [95% CI, −3.6 to −0.7] kg; *P* = .005).

**Table 2.** Primary and Secondary Outcomes

Outcome	Unadjusted change, mean (SD)		Adjusted mean treatment difference (95% CI)^a^	*P* value^a^
	Intervention	Usual care		
**Primary **				
Weight, kg				
12 mo	−2.6 (6.0)	−0.3 (4.2)	−2.1 (−3.6 to −0.7)	.005
24 mo	−1.1 (5.7)	−1.1 (7.1)	0.1 (−1.8 to 1.9)	.93
**Secondary **				
BMI				
12 mo	−1.0 (2.3)	−0.1 (1.6)	−0.8 (−1.4 to −0.3)	.004
24 mo	−0.4 (2.2)	−0.4 (2.7)	0.0 (−0.7 to 0.7)	.99
5% weight loss, No. (%)^b^				
12 mo	22 (25.9)	9 (9.2)	15.3 (5.1 to 26.0)	.003
24 mo	22 (24.2)	15 (15.2)	8.7 (−2.0 to 20.2)	.10
Waist circumference, cm				
12 mo	−4.3 (12.6)	−1.9 (5.4)	−2.3 (−5.1 to 0.5)	.11
24 mo	0.3 (7.1)	−0.1 (7.2)	0.5 (−1.6 to 2.7)	.61
Leisure time moderate and vigorous physical activity total min/wk				
12 mo	35.8 (226.3)	−36.9 (277.5)	33.3 (−27.5 to 94.1)	.28
24 mo	6.4 (291.6)	−74.1 (301)	46.4 (−24.9 to 117.7)	.20
Leisure time physical activity MET min/wk^b^				
12 mo	218.5 (1159)	−231 (1411)	242.6 (−61.3 to 546.5)	.12
24 mo	−34.2 (1343)	−386 (1541)	179 (−147.9 to 505.9)	.28
Total energy expenditure, kcal/kg/d				
12 mo	0.3 (5.0)	−0.4 (3.9)	0.8 (−0.3 to 1.9)	.18
24 mo	0.7 (5.6)	−0.8 (4.3)	1.4 (−0.1 to 2.9)	.06
DASH score				
12 mo	0.4 (1.4)	−0.2 (1.8)	0.2 (−0.2 to 0.7)	.31
24 mo	0.3 (1.8)	−0.2 (2.0)	0.1 (−0.5 to 0.6)	.78
Fruit and vegetable, servings/d				
12 mo	−0.2 (3.1)	−0.9 (4.3)	0 (−1.0 to 1.1)	.95
24 mo	1.1 (4.0)	−0.4 (5.2)	0.6 (−0.7 to 2.0)	.36
Total calorie intake, kcal/d				
12 mo	−168 (760)	−235 (910)	−48.2 (−246.9 to 150.5)	.63
24 mo	−85.2 (671)	−268 (703)	56.5 (−160 to 273.1)	.61
Total fat, g/d				
12 mo	−15.0 (44.6)	−10.2 (50.1)	−6.5 (−17.4 to 4.5)	.24
24 mo	−8.0 (40.9)	−8.7 (41.6)	−0.6 (−12.9 to 11.6)	.92
Obesity-related problem scale				
12 mo	−11.1 (28.0)	−9.1 (23.2)	1.6 (−5.2 to 8.4)	.65
24 mo	−10.0 (23.8)	−8.2 (26.0)	2.1 (−5 to 9.1)	.56
EQ-5D, no symptoms, No. (%)				
Mobile^c^				
12 mo	57 (82.6)	68 (81.0)	1.8 (−11.0 to 10.2)	.84
24 mo	66 (83.5)	74 (81.3)	1.1 (−8.7 to 9.2)	.89
Care^c^				
12 mo	64 (92.8)	78 (92.9)	−0.7 (−0.8 to 0.2)	.46
24 mo	75 (94.9)	82 (90.1)	0.4 (−0.3 to 0.6)	.64
Activity^c^				
12 mo	56 (81.2)	72 (85.7)	−6.7 (−28.3 to 1.1)	.22
24 mo	69 (87.3)	77 (84.6)	0.1 (−10.6 to 6.6)	.98
Pain^c^				
12 mo	32 (46.4)	44 (52.4)	−6.7 (−25.4 to 10.4)	.45
24 mo	38 (48.1)	48 (52.8)	−7.8 (−27.6 to 10.3)	.39
Anxious^c^				
12 mo	41 (59.4)	47 (56.0)	12.2 (−8.8 to 33.5)	.26
24 mo	49 (62)	52 (57.1)	10.0 (−13.1 to 31.9)	.31

Abbreviations: BMI, body mass index (calculated as weight in kilograms divided by height in meters squared); EQ-5D, EuroQol 5-Dimension; DASH, Dietary Approach to Stop Hypertension; MET, metabolic equivalents.

^a^ Mixed-effects models accounting for the random effects of repeated measures and primary care physicians and adjusted for baseline value of the outcome of interest, study site, age, sex, BMI, waist circumference, and Short Acculturation Scale for Hispanics. For Nutrition Data System for Research outcomes (ie, DASH score, fruit and vegetable, total calorie intake, and total fat), the additional variable Mexican origin (yes or no) was adjusted.

^b^ Includes data for 91 individuals in the intervention group and 99 individuals in the usual care group.

^c^ Includes data for 82 individuals in the intervention group and 92 individuals in the usual care group.

A significantly greater proportion of intervention participants achieved clinically significant weight loss (ie, ≥5% of baseline weight) compared with control participants at 12 months (22 participants [25.9%] vs 9 participants [9.2%]; *P* = .003) but not 24 months (22 participants [24.2%] vs 15 participants [15.2%]; *P* = .10). There was no significant effect of the intervention compared with the control on secondary outcomes of waist circumference, leisure time physical activity, total energy expenditure, obesity-related problems, and health-related quality of life at 12 or 24 months (Table 2). We did not detect moderation by any of the prespecified variables.

### Intervention Adherence and Weight Loss

**Figure 2.**
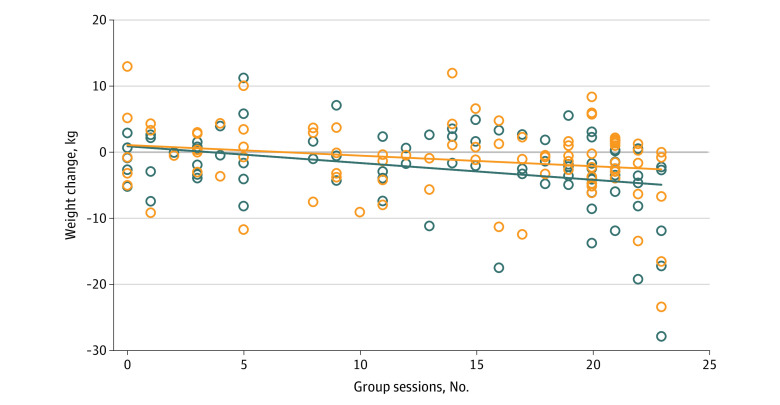
Correlation of Session Attendance and Weight Change

Session attendance was significantly positively correlated with weight loss at 12 months (ρ = −0.33 [95% CI, −0.51 to −0.13]; *P* = .002) but not at 24 months (ρ = −0.21 [95% CI, −0.40 to <0.01]; *P* = .05) (Figure 2). Participants who achieved clinically significant weight loss at 12 months attended more sessions than those who did not (mean [SD], 16.6 [7.6] sessions vs 12.4 [7.5] sessions; *P* = .03). Greater monitoring of weight, diet, and physical activity were associated with greater weight loss (Table 3).

**Table 3.** Percentage of Completions for the Key Intervention Components Associated With Weight Change From Baseline at 12 and 24 Months

Follow-up	Component sessions attended, %								
	Weight monitoring			Diet monitoring			Physical activity monitoring		
	<50 (n = 33)	50 to <75 (n = 22)	≥75 (n = 37)	<50 (n = 70)	50 to <75 (n = 9)	≥75 (n = 13)	<50 (n = 59)	50 to <75 (n = 14)	≥75 (n = 19)
Weight change from baseline, mean (SD) kg									
12 mo	0.1 (3.9)	−2.2 (5.7)	−4.7 (6.6)^a^	−0.9 (4.5)	−8.1 (9.2)^a^	−6.4 (5.7)^a^	−0.4 (4.5)	−5.3 (5.5)^a^	−6.5 (7.2)^a^
24 mo	0.2 (5.1)	−1.3 (6.3)	−2 (5.9)	−0.2 (5.3)	−4.5 (8)^a^	−3.2 (5.1)	0.1 (5.4)	−2.4 (4.7)	−3.5 (6.6)^a^

^a^ Indicates significant difference compared with <50% completion group.

### Outside Weight Management Participation and Adverse Events

There were 19 participants in usual care who participated in various weight management programs, and 9 participants in the intervention who participated in other weight management programs outside this study. Over the 24-month trial, there were 36 serious adverse events, 16 requiring hospitalization and 11 involving fractures or musculoskeletal injuries that required outpatient repair procedures. Of these 36 serious adverse events, 4 (11.1%) were possibly related to the study (ie, hospital admissions following elective panniculectomy and abdominoplasty, torn meniscus, ankle surgery, and finger fracture). There were 202 nonserious adverse events, with 64 (31.7%) involving minor musculoskeletal injuries being the most common and 12 of 64 minor musculoskeletal injuries possibly related to the study. Combined serious adverse events (14 events in the intervention group vs 22 in the control group) and nonserious adverse events (101 events in the intervention group vs 101 events in the control group) were comparably distributed across both groups. There were no deaths.

## Discussion

In this randomized clinical trial, Latino primary care patients randomized to a culturally adapted, technologically mediated behavioral lifestyle intervention did not lose more weight at 24 months than those randomized to usual care. However, participants in the intervention lost more weight and were more likely to achieve clinically significant weight loss (ie, ≥5%) at 12 months than those randomized to usual care. There were no significant effects of the intervention on secondary outcomes at 12 or 24 months. Increased session attendance was significantly associated with achieving clinically significant weight loss at 12 months.

In the only other randomized clinical trial of a culturally adapted diabetes prevention intervention with 24 months of follow-up, to our knowledge, there was also no difference in weight loss between the intervention and usual care groups.^16^ Both studies provided minimal contact with participants during the second year. It is possible that participants need additional support in the second year to continue to lose weight or sustain weight loss, as has been done in previous trials, albeit not with primarily Latino adults.^52,53,54^

The range of weight loss observed over 12 months in this study and in other culturally adapted interventions (ie, 1.1 kg to 4.2 kg) is less than what was observed in the original DPP trial and behavioral weight loss interventions in primary care.^11,15,16,17,18,19,20,55^ For instance, in a study of 243 primary care patients (78% non-Hispanic White patients), those randomized to receive the Group Lifestyle Balance lost 6.3 kg over 15 months, which was significantly greater than usual care.^51^ It is unknown whether lower levels of weight loss are owing to the cultural adaptation or to barriers that Spanish-speaking Latino adults face in achieving weight loss.

As has been documented in prior studies,^54,56,57,58^ session attendance was positively associated with weight loss. Technology-mediated strategies, such as online videos or videoconference sessions, could be incorporated to increase session attendance. In a study of 351 primarily African American primary care patients in North Carolina, patients received a study-specific smartphone application and 18 telephone calls from a coach. More than two-thirds of participants randomized to the intervention completed 80% or more of the coach telephone calls and lost 5.2 kg over 12 months.^22^

### Limitations

This study has some limitations. There was insufficient power to detect whether moderation was present. Understanding possible heterogeneity of effects is critical for informing future strategies to optimize effectiveness. Additionally, the findings of this study may not apply to other Latino populations. Latino adults in this study were recruited from a health system accessed primarily by patients with employer-based health insurance and as a result had relatively high annual incomes compared with other similar studies with Latino adults. Thus, the findings of this study may not be generalizable to Latino adults with different socioeconomic profiles. Additionally, this translational study was not designed to examine mechanisms, such as change in diet and physical activity. As a result, more rigorous measures of dietary intake and objective measures of physical activity were not used.

## Conclusions

This randomized clinical trial found that a technology-mediated culturally adapted behavioral lifestyle intervention was effective for weight loss over 12 months but not 24 months among adult Latino primary care patients at risk for diabetes. Identified factors associated with weight loss revealed opportunities for future research to test strategies to optimize effectiveness of behavioral lifestyle interventions with Latino adults. Given that Latino individuals are disproportionately represented among adults in the US with obesity and diabetes, it is critical to pursue future research aimed at increasing effectiveness of behavioral lifestyle interventions for chronic disease prevention.

## Notes

Supplement 1. **Trial Protocol**

Supplement 2. **eTable 1.** Sensitivity Analysis of Primary Outcome Using Study-Measured Weights **eTable 2.** Sensitivity Analysis Using Bootstrap Results for the Treatment Effects on Weight Changes From Baseline at 12 and 24 Months

Supplement 3. **Data Sharing Statement**
