# Supplementary material for: Effect of a Culturally Adapted Behavioral Intervention for Latino Adults on Weight Loss Over 2 Years: A Randomized Clinical Trial
Source: JAMA Netw Open. 2020 Dec 18;3(12):e2027744. doi: 10.1001/jamanetworkopen.2020.27744 (PMC7749441; doi:10.1001/jamanetworkopen.2020.27744)
Supplement: Supplement 1. — Trial Protocol [file jamanetwopen-e2027744-s001.pdf]

Evaluation of a culturally-adapted lifestyle intervention to treat elevated cardiometabolic risk of Latino adults in primary care (Vida Sana): A randomized controlled trial

Author names and affiliations:

Lisa G. Rosas[a], Nan Lv[a], Lan Xiao[a], Megan Lewis[b], Patricia Zavella[c], M. Kaye Kramer[d], Veronica Luna[a], Jun Ma[a,e]

[a]Palo Alto Medical Foundation Research Institute
795 El Camino Real, Ames Building, Palo Alto, CA 94301
rosasl@pamfri.org
[b]RTI International
3040 Cornwallis Rd., PO Box 12194, Research Triangle Park, 27709
melewis@rti.org
[c]University of California
1156 High Street
Santa Cruz, CA 95064
Zavella@ucsc.edu
[d]University of Pittsburgh
3512 Fifth Avenue
Pittsburgh, PA 15213
mkk3@pitt.edu
[e]University of Illinois at Chicago
Chicago, IL 60607
maj2015@uic.edu
Corresponding author: Lisa G. Rosas

Target journal: Contemporary Clinical Trials

Abstract

Latinos bear a disproportionate burden of the dual pandemic of obesity and diabetes. However, successful interventions addressing this disparity through primary care are lacking. To address this gap, the 5-year Vida Sana (Healthy Life) study tests a culturally adapted and technology-enhanced group-based Diabetes Prevention Program intervention in a randomized controlled trial with overweight/obese Latino adults who have metabolic syndrome and/or pre-diabetes. Eligible, consenting patients (n=186) from a large community-based multispecialty group practice in Northern California will be randomly assigned to receive the culturally-adapted intervention or usual care. The RE-AIM (Reach, Effectiveness, Adoption, Implementation, and Maintenance) framework guided the planned evaluations. The primary aim is to determine the effectiveness of the intervention (the "E" in RE-AIM). We hypothesize that the intervention will lead to a greater mean reduction in weight at 24 months (primary endpoint) vs. usual care. Secondary outcomes will include measures of cardiometabolic risk factors (e.g., blood pressure), psychosocial well-being (e.g., health-related quality of life), and behavior change (e.g., physical activity). The secondary aim is to evaluate the other RE-AIM dimensions using mixed methods: reach (e.g., participation rate of the target population), adoption (e.g., participating clinic and provider characteristics), implementation (e.g., intervention fidelity), and maintenance (e.g., sustainability in the practice setting). These findings have real word applicability with value to clinicians, patients, and other decision makers considering effective diabetes prevention programs for primary care that would support the millions of Latino adults who experience a disproportionate burden of diabetes.

Word count: 240

Trial registration: NCT02459691

1. Introduction

Ranking as the largest and fastest growing minority group in the United States (US), Latinos reached 55 million in 2014.[1] The prevalence of overweight and obesity is higher among Latino adults (77%) than non-Hispanic whites (68%).[2] Consequently, Latinos have a higher incidence of type 2 diabetes and prevalence of major cardiovascular risk factors (e.g., metabolic syndrome, pre-diabetes).[3-7]

Previous studies have shown that behavioral lifestyle interventions are effective for promoting modest yet clinically significant weight loss and can delay or prevent the onset of diabetes in high-risk adults in community and primary care settings.[8-11] For example, the Diabetes Prevention Program (DPP) trial showed an intensive lifestyle intervention targeting modest weight loss (7%) and increased physical activity (150 minutes per week) lowered type 2 diabetes incidence by 58% among high-risk, multiethnic adults (55% non-Hispanic whites, 20% black, and 16% Hispanic/Latino).[12] Follow-up data showed that the intervention benefits persisted for at least 10 years.[13] To promote dissemination, the original, resource intensive, primarily one-on-one curriculum was adapted to a group program with fewer sessions, called Group Lifestyle Balance (GLB).[14-16] The one-year GLB curriculum is approved by the Centers for Disease Control and Prevention through the national Diabetes Prevention Recognition Program[17] and has been proven to be feasible and effective in community and primary care settings.[14,18-22]

Technology has potential to increase the reach, effectiveness, and scalability of behavioral lifestyle interventions such as the GLB.[23-32] We previously demonstrated the effectiveness of supplementing the GLB 12-session core curriculum with technology-mediated lifestyle coaching via secure email messaging and web-based self-monitoring of weight and physical activity to reduce obesity and cardiometabolic risk factors in a primary care setting in the E-LITE (Evaluation of lifestyle interventions to treat elevated cardiometabolic risk in primary care) study.[33-38] However, similar to other rigorous lifestyle intervention trials in primary care,[8,39,40] Latinos were <5% of total participants. Communication technologies (e.g., web, email, mobile) and wearable devices (e.g., pedometers, accelerometers) offer opportunities to tailor interventions to diverse subgroups such as Latinos as well as to promote effectiveness by adapting to individuals' response to interventions over time.[41,42]

The primary care setting is ideal given opportunities for primary care physicians to refer at-risk patients, provide on-going management for comorbidities, and support maintenance of preventive lifestyle behaviors. For Latinos, increasing access to healthcare as a result of the Affordable Care Act (for those eligible for insurance or with insurance),[43] makes primary care-based programs increasingly advantageous. Additionally, behavioral lifestyle interventions based in primary care provide an opportunity to provide healthcare for Latinos that is personal, welcoming and concerned for the individual in a social context, which is favored by Latino cultural values.[44-46]

To date, few effective and practical behavioral weight-loss interventions that leverage technology and are based in primary care have been developed and tested among high-risk Latinos: a large, vulnerable population with persistent health disparities. To fill this critical gap, the Vida Sana (Healthy Life) study was designed to evaluate a culturally-adapted, technology-enhanced intervention targeting overweight or obese Latino adults with pre-diabetes, a history of gestational diabetes, and/or metabolic syndrome in a community primary care setting.

2. Methods

2.1. Study Design

This pragmatic RCT (11/2014-08/2019) will evaluate a culturally-adapted intervention based on the Group Lifestyle Intervention among high-risk Latino adults. For the purposes of this study, 'Latino' refers to people who self-identify as Latino or Hispanic. The culturally adapted intervention was developed through rigorous formative research and pretesting by a Latino Patient Advisory Board. The specific aims focus on the primary outcome of weight and the evaluation of the domains of the RE-AIM (Reach, Effectiveness, Adoption, Implementation, and Maintenance) framework:[47]

Aim 1: Compare the culturally-adapted intervention and usual care for overweight or obese Latino adults with pre-diabetes and/or metabolic syndrome, but without diabetes or cardiovascular disease (the E in RE-AIM).

Hypothesis 1: Compared with controls, intervention participants will achieve a greater mean reduction in weight from baseline to 24 months (primary outcome).

Hypothesis 2: Compared with controls, intervention participants will achieve greater improvements in secondary outcomes including measures of cardiometabolic risk factors (e.g., Body Mass Index, waist circumference, and blood pressure), psychosocial well-being (e.g., health-related quality of life), and lifestyle behaviors (e.g., diet, physical activity).

Aim 2: Assess intervention attributes in the other RE-AIM domains to gauge generalizability and guide future implementation.

We will use mixed methods to measure other four RE-AIM attributes: Reach, Adoption, Implementation, and Maintenance; and conduct subgroup and effect mediation analyses to explore which patients benefit more and how so.

2.2. Pragmatic RCT

2.2.1. Eligibility Criteria

We will apply permissive inclusion criteria and minimally necessary exclusion criteria to optimize the balance between generalizability, patient safety, intervention adherence, and retention. We will enroll Spanish-speaking or bilingual Latino adults $\geq$ 18 years with a BMI $\geq$ 24 kg/m^2 ($\geq$ 22 if of Asian ancestry) and pre-diabetes,[48] a history of gestational diabetes, and/or metabolic syndrome,[49] but without type 1 or type 2 diabetes or cardiovascular disease (**Table 1**) who are active patients at the Palo Alto Medical Foundation (PAMF), a large community-based multispecialty group practice in Northern California. Patients with significant psychiatric (e.g., bipolar or psychotic disorder) or medical comorbidities (e.g., active cancer, organ failure) will be excluded. Additional exclusions are to protect participant safety (e.g., pregnancy) and prevent loss to follow-up (e.g., planned relocation, limited lifespan).

2.2.2. Recruitment and Screening

The targeted enrollment of 186 participants will be met in three sequential cohorts of 62 patients each. Each cohort will be recruited from one of three different clinic sites within PAMF where participants randomly assigned to the intervention group will attend the group sessions. Recruitment and screening will proceed in four steps. First, PAMF patient EHRs will be pre-

screened to identify potential participants meeting basic eligibility criteria (e.g., age, active patient status, and absence of exclusionary medical or psychiatric comorbidities). Second, Primary Care Physicians (PCPs) at each site will review lists of potentially eligible patients, exclude those they deem inappropriate for the study because of medical reasons, and authorize study contact for the rest. Third, PCP-approved patients will receive a recruitment email or letter in Spanish and English introducing the study and inviting them to complete an initial brief screening online, which focuses on those eligibility criteria that individuals can reliably assess themselves (e.g., pregnancy, likelihood of relocation). Two weeks after sending the email or letter, recruitment staff will phone patients who have not done self-screening and who did not opt out, to complete the initial screening. Fourth, patients who screen eligible will complete an in-person baseline visit at the clinic site from which they were recruited. Prior to the visit, patients will receive the link to a self-administered survey for completion prior to the in-person visit so as to reduce the overall time of the visit. If patients cannot or do not want to complete the survey prior to the visit, they can do so at the visit. The visit will begin with obtaining written informed consent. A trained bilingual research assistant will orally administer the baseline questionnaire and conduct standardized height, weight, waist circumference, and blood pressure measurements.[50-52]

2.2.3. Randomization and Blinding

Eligible participants will be randomized in a 1:1 ratio to receive usual care or usual care plus the intervention (n = 93/arm). We will apply a covariate-adaptive biased coin method that we have published[53] and used successfully in several trials[54,55] to achieve good marginal balance between treatments across the following baseline characteristics: clinic, age, sex, BMI, waist circumference, and level of acculturation assessed by the Short Acculturation Scale for Hispanics.[56,57] The dynamic block randomization algorithm of our method automatically ensures allocation concealment. By design, treatment will be identifiable to participants and the lifestyle coach, but masking of the investigators, Data and Safety Monitoring Board, outcome assessors, and data analyst will be enforced. The bilingual and bicultural lifestyle coach will be masked to participants' official study measurements, but not their self-measurements tracked on MyFitnessPal.

2.2.4. Continuation of Usual Care

For patient safety and generalizability, no standard care will be withheld at any time after enrollment. We will recruit from patients who have used PAMF for routine care for $\geq$ one year and thus have a higher likelihood of establishing a relationship with their PCP. Regardless of treatment assignment, participants will not be restricted from seeking weight loss treatment from their physician or in the community, to control for changes in medical practice and secular trends and to protect external validity. From the EHR we will determine PCP orders and referrals throughout the trial period for intervention and control participants. We will also survey participants about any programs or products that they may have used on their own to treat obesity during the trial. We will conduct secondary analyses using data on out-of-study obesity treatments to elucidate their potential confounding effects on the primary intention-to-treat (ITT) findings. Based on EHR and survey data collected in E-LITE, 15 of the 81 controls used an out-of-study weight loss program (13 used a commercial and two used a PAMF program), compared with five of the 79 coach-led and three of the 81 self-directed participants (all used a commercial program), during the 15-month trial period (P = .003). No one underwent pharmacological or surgical weight loss treatment. These data suggest a low participation rate

in non-study programs among research participants; to the extent that it occurred in E-LITE, it supported the robustness of the primary ITT findings.[37]

2.2.5. Intervention and Fidelity Assurance

In addition to usual care, intervention participants will receive a culturally-adapted intervention facilitated by a trained bilingual/bicultural coach. The intervention sessions will take place at the clinic site where the patient was recruited.

2.2.5.1. Theoretical basis

The intervention is based on Social Cognitive Theory,[58] which emphasizes a triadic, reciprocally deterministic relationship between the individual, environment, and behavior. It recognizes that behavior change is a dynamic process that moves at variable speed through stages of readiness to change. Positive outcome expectancies through realistic goal setting and guided action planning are associated with initiation of behavior change. Self-efficacy developed for specific behaviors (e.g., physical activity) predict establishment and maintenance of behavior change. Social Cognitive Theory suggests that self-efficacy is enhanced through social support and gradual mastery of self-regulation skills (e.g., goal setting, self-monitoring).[59]

2.2.5.2. Vida Sana format and lifestyle coach

The Vida Sana intervention is a cultural adaptation of the original GLB. The program is delivered in Spanish with Spanish-language handouts and utilizes Smartphone and web applications that are available in Spanish. A bilingual and bicultural lifestyle coach with a bachelor's degree who undergoes standard training with a GLB master trainer (see 2.2.5.5) delivers the intervention. Information about the 2-day GLB standard coach training can be found on the website of the University of Pittsburgh Diabetes Prevention Support Center.[16] The lifestyle coach does not need additional training or advanced degrees to serve in this role.

As in the original GLB curriculum,[16] the Vida Sana intervention has two distinct components: 1) intensive treatment (core) and 2) post-core support. The intensive treatment component includes 12 weekly core sessions followed by four bi-weekly core transition sessions, for a total of 16 sessions delivered within the first six months of the program. It uses a goal-based approach to promote positive outcome expectancies and foster self-efficacy by targeting at least 7% weight loss and a minimum of 150 minutes per week of moderate-intensity physical activity. Moderate caloric reduction by 500-1,000 kcal/day through healthy substitutions and portion control, rather than omission or elimination of specific foods, is recommended.[50] The post core support phase includes an additional six sessions delivered monthly over the course of six months for a total intervention duration of 12 months. Post core support phase focuses on (1) facilitating continued behavior change through an iterative guided mastery process;[60] (2) fostering participants' self-efficacy and independence; and (3) reinforcing problem-solving and behavior maintenance skills. A healthy meal, often a healthy version of a traditional dish, is provided at each session.

Formative research and pretesting by a Latino Patient Advisory Board informed the cultural adaptations for Vida Sana. The Vida Sana intervention builds upon references to family in the original GLB and includes three opportunities to include family members during the in-person group sessions. First, Vida Sana includes a family-wide orientation session prior to Session 1. This provides an opportunity for the lifestyle coach to describe the Vida Sana intervention to the family and to provide family members with concrete strategies for demonstrating emotional,

structural, and informational support to the participant. In addition, families are encouraged to engage in fun activities to promote a positive family environment that is supportive for lifestyle changes. Second, the lifestyle coach encourages participants to invite one family member to Session 6: “Take Charge of What is Around You.” This session focuses on modifying the physical and social environment to promote healthy behaviors. Family members are engaged to work with the participant to identify and make these changes. Third, participants are encouraged to bring a family member to session 12 after which the frequency of the sessions reduces to bimonthly and monthly. At session 12, family members are encouraged to provide support during this transition. In addition to the emphasis on family, the Vida Sana intervention includes other modifications that were recommended by the Latino Patient Advisory Board. For example, the MyPlate[61] graphic and recommendations is introduced in session 1 as opposed to session 3 in the original GLB. Similarly, the physical activity monitor is introduced in session 1 as opposed to session in 4 in the original GLB. These additions to session 1 were made possible by the addition of the family-wide orientation session that covered information on the program that was originally covered in GLB session 1. Other modifications were primarily superficial changes to example foods or example situations.

2.2.5.3. Technology-enhanced coaching and self-monitoring

Based on the success of the E-LITE trial,[37,38] the culturally-adapted intervention incorporates self-monitoring via MyFitnessPal on a mobile device or computer, a wireless physical activity monitor (FitBit), and individualized feedback through the Smartphone application. Self-monitoring is key to success in behavioral weight-loss interventions.[62] Participants are encouraged to track their weight, diet and physical activity on a daily basis through the MyFitnessPal application, which is available in Spanish. Pedometer-measured daily steps are captured via the FitBit activity monitor and automatically synched with the FitBit Smartphone or web application. The coach reviews participants’ self-monitoring data and provides individualized feedback via the Smartphone application every week during the core phase and in response to participants’ needs in the post-core phase. The coach provides feedback and counseling on actionable lifestyle change and problem-solving strategies (e.g., to reduce intake of fat and calories) to help participants reach their goal of 7% weight loss and 150 minutes of physical activity. Additionally, for 12 months following the intervention, the coach sends monthly messages to all participants reinforcing intervention topics and offering support for maintenance of lifestyle behaviors. This form of coach-participant interaction is intended to support the participant in tailoring the implementation of recommended lifestyle change strategies to achieve feasibility and sustainability within their own home and social environments, and to aid in problem solving and relapse prevention. The coach focuses on dietary change, physical activity, and behavioral skills training suited to what each participant is eating and doing, and the changes (s)he is willing and able to make given his or her own resources and supports. These added features will likely enhance the proposed intervention’s reach and adoption potential given the prevalence of online and mobile technology use among US Latinos.[63]

2.2.5.4. Fidelity assurance

We will follow recommendations for quality assurance in behavioral interventions.[64] Use of standardized intervention materials, structured staff training and ongoing oversight are fundamental to ensuring high intervention fidelity. The lifestyle coach will undergo standardized training by a certified GLB master trainer with supplemental training on the cultural adaptations resulting from the formative research. Per our standard practice, all group sessions will be audiotaped and a random 10% sample from the sessions by recruitment cohort will be audited and graded using a session-by-session rating scale from a previous trial.[37] The coach will complete a checklist of critical intervention behaviors and materials delivered during each

session. Self-monitoring records and Smartphone application communication are readily retrievable and will be reviewed as part of routine quality control efforts. Falling below an a priori performance standard (e.g., 90% adherence to intervention protocol) will trigger more frequent audit and feedback and, if needed, “booster” training for the coach. Participant engagement and adherence are also essential to intervention fidelity and must be monitored and supported. Participant progress on key intervention tracking parameters (e.g., date, format, duration of contact, most current weight, and physical activity level) will be routinely documented. The coach will review and give feedback on homework and self-monitoring records and document participant progress toward protocol-specific, achievement-based objectives. She will routinely inquire about barriers to intervention receipt and adherence, recommend personalized, actionable problem-solving strategies, and provide ongoing support via proactive follow-up.

2.2.6. Participant Safety

PCP approval will be required before potentially eligible patients are contacted by the study. Participants will be carefully screened and individuals for whom the interventions would be medically inappropriate or unsafe are excluded. During screening, women who are pregnant, lactating, or planning to become pregnant during the study period are excluded. If a participant becomes pregnant during the study, she is excluded immediately from further participation in all study activities, and her PCP is immediately notified. Participants who develop any other exclusionary condition (e.g., diabetes) following randomization may continue with the interventions and follow-up assessments with their PCP’s approval. To ensure unbiased ascertainment between the intervention and control group, outcome assessors will systematically screen all participants for adverse events during in-person assessments at baseline, 12, and 24 months using a standard interview and reporting form as done in our previous trials.[33,55,65,66] In addition, outcome assessors will call all patients at six and 18 months to screen for adverse events. Positive responses trigger an adverse event record, which is reviewed by the study physician for seriousness, study relatedness, and expectedness. Similar information reported by participants at other times (e.g., during intervention encounters) is duly noted and followed up with, as needed, to assure participant safety. Participants will be referred to their PCP for a medical evaluation and follow-up as needed or recommended by the study physician. We will report adverse events according to the data and safety monitoring plan (**Appendix A**).

2.2.7. Retention

As we have done in our previous trials,[33,55,65,66] we will maximize adherence and retention by careful selection and training of staff, systematic quality control, and adhering to high-quality practices to maintain subject participation in the study. We will use a tracking database to facilitate coordination and monitoring of participant-level activities. No individuals will be randomized without eligibility verification or complete baseline data. Examples of processes that facilitate retention at follow-up include thorough and fully informed roles and responsibilities of staff and participants, conveying an appreciation of participation and study identification, nominal remuneration for study visits, reasonable accommodations to participant schedules, and prudent participant incentives (pedometer and cash incentives). We will contact participants who miss a visit to reschedule and to re-engage them in subsequent follow-ups. Using a combination of these strategies, we have consistently achieved high retention in several RCTs of similar scope,[37,54,67] including those with Latino participants.[68,69]

2.2.8. Study Measures and Data Collection Schedule

Assessments will occur at baseline, 12, and 24 months on clinical, behavioral, and psychosocial outcome measures at one of the PAMF clinics from which participants are recruited **(Table 2)**. These include the following primary and secondary outcomes and potential effect modifiers and mediators.

2.2.8.1. Primary and secondary outcomes (Aim 1)

Primary outcome, weight, will be assessed according to standard protocols.[52] Secondary outcomes include measures of cardiometabolic risk factors (e.g., BMI, waist circumference, and blood pressure), psychosocial well-being (e.g., health-related quality of life), and behavior change (e.g., diet, physical activity). Trained bilingual research assistants will conduct anthropometric and blood pressure measurements,[50-52] and multiple-pass 24-hour dietary recalls.[70-72] Online self-administered questionnaires and in-person interviewer-administered questionnaires (see **Table 2**) will be used to assess additional secondary outcomes including physical activity,[73] health-related quality of life,[74] obesity-specific quality of life,[75] depressive symptoms,[76,77] and sleep habits and quality.[78] All these surveys have been validated in English and Spanish. Additionally, we will abstract data from the EHR on laboratory values (e.g., HbA1c, fasting glucose, lipid levels), medication prescriptions, and health care encounters for 24 months before and after randomization.

2.2.8.2. Potential effect modifiers and mediators (Aim 2)

To complement the primary and secondary outcomes, we will explore for whom and under what condition (effect modifiers) and how (effect mediators) treatment effects occur. Potential modifiers include sociodemographics (e.g., age, sex, education, employment, occupation, marital status, household size, income, country of origin), food insecurity,[79] acculturation,[80] and health literacy.[81] Potential mediators include self-efficacy,[82,83] social support,[84] and intervention adherence. Intervention adherence will be tracked using session attendance, self-monitoring data, and communication between the participant and coach.

2.2.8.3. Process evaluation (Aim 2)

To help contextualize the effectiveness evaluation under Aim 1, we will conduct a detailed process evaluation with mixed methods to gain a nuanced understanding of why the intervention is (or is not) superior to usual care, whether high intervention fidelity is achieved, what barriers and enablers there are, how these may translate into future implementation, and what modifications can maximize implementation success.[85] We frame the process evaluation around the RE-AIM framework's reach, adoption, implementation, and maintenance domains (**Table 3**).[86-88] We will conduct in-depth interviews with patients, recruitment staff, intervention staff, physician champions, and community stakeholders at baseline, study mid-point, and end point according to each RE-AIM component. The interview guides will be adapted from guides that were developed for the same purpose in another ongoing trial.[89]

2.2.9. Statistical Analysis

2.2.9.1. Analytical plan

The primary hypothesis that intervention participants will achieve and maintain lower weight at 24 months than controls will be tested in a repeated-measures mixed model (Aim 1).[90-92] Secondary hypotheses are analogous, but with different outcome variables, and will be tested using repeated-measures, mixed-effects, linear (for continuous variables) or logistic models (for discrete variables).

$$\mathbf{Y_t = \beta_0 + \beta_1 X + \beta_2 Y_0 + \beta_3 T + \beta_4 (XT) + \Sigma\beta_{4+i} Z_i + \alpha + \gamma + \varepsilon} \qquad \mathbf{(1)}$$

Let Y_t be participants' post-randomization values of the outcome variable at visit T (12 or 24 months). Given the covariate-adaptive randomization, distributions of baseline values on the outcome variable (Y_0) and randomization balancing factors (Z_i) (clinic, age, sex, BMI, waist circumference, and level of acculturation) should be similar between study arms (X) and thus not bias the results. But to the extent they are associated with the outcome, their inclusion in the model will account for otherwise unexplained variation and hence increase efficiency;[93] α and γ are random effects due to clustering of patients within physicians and, in turn, physician within clinics, and patients with intervention classes. The random error, ε, accounts for the non-independence of repeated measures using a covariance structure within participants to be determined by the least Bayesian information criterion. The primary analysis will follow ITT principles and use all available follow-up data, with missing data handled directly through maximum likelihood estimation in mixed modeling. We will document the extent and pattern of missing data and the reasons, and will conduct sensitivity analyses of the impact of missing data on stability of the primary results. For example, we may use available weights up to the point after which data are no longer available (e.g., dropouts) or should not be used (e.g., pregnancy) and then employ multiple imputation[94,95] based on a predictive distribution for future weights with the mean possibly adjusted depending on the pattern and extent of missing data. We will verify that mixed model-based results are not sensitive to violations of model assumptions with permutation and bootstrap resampling tests.[96,97]

Subgroup analyses of pre-specified potential modifiers (**Table 2**) of the intervention effect on weight change will be performed by expanding equation 1 to include the appropriate modifier-by-study-arm interactions. Testing whether the β coefficients of the interaction terms are equal to zero is equivalent to testing the null hypothesis that the subgroup of interest does not independently modify the intervention effect.

Longitudinal (e.g., change in mediator from baseline to 12 months and change in primary outcome from 12 to 24 months) and contemporaneous (e.g., changes in mediator and outcome from baseline to 24 months) mediation will be examined separately by MacKinnon's product of coefficients test (αβ).[98] Asymmetric confidence limits will be constructed based on the distribution of the product with the PRODCLIN program.[99] Because multi-collinearity may be present in multiple mediator models, we first will test each mediator in single-mediator models. Multiple-mediator models including all variables that are at least marginally significant in the single-mediator models will test for independent and suppression effects. To determine the extent of mediated effect, the percentage of total effect mediated will be calculated for each significant mediator as $\alpha\beta/(\alpha\beta+\gamma)$, where γ is the direct intervention effect on outcome. The effect modification-mediation analyses are hypothesis generating only, but we pre-specify the variables to ensure a focus.

We will analyze the quantitative process data using standard tests (e.g., Student's *t*-tests for continuous variables and χ^2 tests for categorical variables). These analyses will be descriptive and not inferential. We will transcribe, code, and analyze qualitative data using Atlas.ti.[100] We will develop a codebook of codes and definitions based on the RE-AIM domains assessed, and use it to train coders and guide data coding. To identify themes we will use content analysis methods.[101] We will triangulate data from different sources to increase the validity of the qualitative data and to draw conclusions about reach, adoption, implementation, and maintenance of the intervention that would guide future implementations.[102]

2.2.9.2. Sample size and data interpretation

We power this trial on change in weight in kg from baseline to 24 months (primary endpoint). To estimate power, we use a *t*-test with simplified assumptions that compares (24m - baseline) differences between study arms at $\alpha = 5\%$ (2-sided). Actual power may be greater due to increased efficiency associated with repeated-measures mixed models with baseline and covariate adjustments.[103] A sample of 93/arm has 80% power to detect a net between-treatment *M* (SD) difference of 2.1(4.6) kg, assuming up to a 20% loss to 24 months of follow-up. As a conservative estimate, this effect size is based on the net weight change in the E-LITE self-directed intervention (corresponding to a mean of 4.5kg, 5.0% weight loss vs. 2.4kg, 2.6% in usual care), which was significantly smaller than that of the coach-led group (6.3 kg-; 6.6% weight loss). Weight loss ≥5% is widely regarded to be clinically significant,[104] whereas a weight change <3% has been used to define weight maintenance.[105] Hence, the net weight change for the E-LITE self-directed intervention relates to the minimal clinically important difference in weight reduction. To preserve statistical power, no multiplicity adjustment will be made for secondary analyses. These analyses are not intended to produce clinically actionable results, but to supplement conclusions based on the primary analysis, and to inform future research. They will be interpreted properly within that context, considering the totality of evidence available.[106,107]

2.2.10. Data Management and Quality Control

All study data will be entered into computerized data files utilizing: (1) Microsoft ACCESS for data entry on recruitment, follow-up, and intervention tracking; (2) REDCap[108] hosted at the PAMF Research Institute for self- and interviewer-administered questionnaire data and physical measurements; (3) the Nutrition Data System for Research (NDS-R) (Minneapolis, MN) licensed for data collection and nutrient analysis based on multiple-pass 24-hour diet recalls;[71,72] and (4) a custom-designed web application for seven-day physical activity recall. All of the data entry systems will employ automatic, real-time range, logic, and missing value checks. Also, the outcome assessors are trained on data collection protocols (e.g., multiple-pass 24-hour diet recall using NDS-R and 7-day physical activity recall), and their performance is continuously monitored. Data sets will be cleaned, verified and archived, and then read into SAS (version 9.2, SAS Institute, Cary, NC) data sets, which also will be archived. One official copy of all the study data and a master data dictionary will be maintained and updated regularly by the study data analyst. All analytic and tracking databases will be stored in a password-protected, encrypted network drive with continuous backups. For the protection of participant confidentiality, unique anonymous study IDs will be used for data storing, tracking and reporting. Protected health information will be stored separately from all other study data, and will be used and disclosed in accordance with the Health Insurance Portability and Accountability Act regulations. Regular reports will be produced on (1) patient accrual and follow-up completion/retention in relation to goals and timeline; (2) the randomization process and group comparability on the balancing variables; (3) key baseline characteristics of the sample, by (blinded) group, related to the primary and secondary outcome variables and proposed effect modifiers and mediators; (4) intervention exposure and adherence; and (5) protocol violations. Any observed delays in these processes or data irregularities will be followed up and resolved in a timely manner.

3. Discussion

The Vida Sana study will provide robust evidence of the effectiveness and potential for implementation and dissemination of a culturally-adapted behavioral lifestyle intervention

incorporating technology and based in primary care for adult Latinos with high cardiometabolic risk. Overweight and obese patients with pre-diabetes and/or metabolic syndrome are a critical group because of their increased lifetime risk for diabetes and cardiovascular disease and the potential reversibility of their condition. Efficacy trials such as the DPP[12] have shown that intensive lifestyle intervention results in significant weight loss and reduced diabetes risk. Yet few DPP translation studies based in primary care and leveraging technology have specifically targeted Latino populations,[11,109-113] a group with higher prevalence of overweight and obesity[3] and higher burden of metabolic syndrome and diabetes than non-Hispanic whites.[5,114]

Among studies focused on Latinos, one RCT was conducted with Latino adults primarily of Caribbean descent who participated in a 1-year community-based, culturally-adapted DPP intervention that included 13 group sessions and three individual home visits.[111] Compared with usual care, intervention participants in that study achieved significantly greater weight loss (median [95% conference interval], −2.5 [−4.0, −1.5] vs 0.63 [−1.05, 2.00] lb; P=.04) and improvement in HbA1c (−0.10 [−0.15, −0.06] vs −0.04 [−0.08, −0.002] %; P=.009) and homeostasis model assessment of insulin resistance (−0.36 [−0.64, −0.09] vs −0.06 [−0.57, 0.38]; P=.03).[112] Another RCT examined the effectiveness a behavioral lifestyle intervention that was adapted from the DPP and included 15 groups sessions over 24 months and four one-on-one case management visits among Latino adults of primarily Mexican descent (n=204). The three-arm study compared the intervention with and without community health worker support (7 visits over 24 months) with usual care in a community health center setting. The interventions were not more effective than usual care according to the primary outcome of change in weight at 24 months.[115]

The Vida Sana study specifically addresses the gap in evidence and health services for obesity management and type 2 diabetes prevention among high-risk Latino adults in primary care settings. Primary care is an ideal setting for behavioral lifestyle interventions. Primary care providers' influence can be leveraged to motivate patients to initiate behavior change, manage issues that arise during engagement, and support maintenance. Such primary care-based interventions also provide healthcare systems opportunities to support culturally centered care for Latinos whose cultural values tend to favor care that is relationship-based and involves more time with patients than office visits generally allow.[44-46] This is because behavioral lifestyle interventions, such as the one being tested in this study, utilize a lifestyle coach as part of the care team that can spend more time with each patient than primary care providers are able to do.

The Vida Sana intervention fuses a traditional, effective delivery modality—group visits—with existing, rapidly expanding health information technology modes of communication (e.g., Smartphone applications, Web-based application, secure e-messaging) to provide culturally and linguistically appropriate obesity management for high-risk Latino adults in primary care. Harnessing the potential of these technologies offers two primary benefits including maximizing intervention effectiveness and reach. First, individualized feedback based on self-monitoring data offers important opportunities for tailoring intervention strategies to the diversity within Latino culture. Second, technologies offer the potential for highly scalable and exportable intervention strategies that can be disseminated in diverse clinical and public health settings. Despite the evidence that internet and mobile phone interventions have shown promise for weight loss and maintenance in adults,[25,27-30,116,117] none of the previously reported studies of DPP translations in Latinos have incorporated technology. Although Latinos historically experienced the 'digital divide,' their access to technology in general and Smartphones in particular make this a particularly promising approach to maximize reach in this population.[118]

The Vida Sana study will fill an important gap in the literature by integrating health information technology with traditional care models (e.g., group visits) to combat obesity among Latinos.

Limitations of the Vida Sana study relate to generalizability to Latinos who do not have access to primary care and technology. This study was specifically designed to address the lack of diabetes prevention lifestyle interventions in primary care settings. Latinos can increasingly benefit from the advantages of the primary care setting given the increasing numbers of Latinos gaining health insurance and access to care as a result of Affordable Care Act and Medicare expansion.[43] The Vida Sana trial will provide critical evidence to support culturally-centered behavior therapy for obesity and diabetes prevention in primary care settings as Latinos gain increasing access over time. Similarly, current national data show that Internet and mobile technology access and use for health are comparable, and sometimes even greater, in Latinos than in non-Hispanic whites.[119,120] In 2015, 50% of US Latinos (vs. 72% white) have broadband Internet access, 71% (vs. 61%) have smartphones, and 73% (vs. 58%) use these technologies for searching health information.[120-122] Also, emerging studies find Latinos can effectively use technology tools to improve health behaviors such as physical activity.[123,124] Continued penetration of technology is expected. That a segment of the Latino population does not currently have access to the Internet and/or mobile technology should not be a barrier to developing the evidence base for health interventions using these technologies, which, if not addressed, would only accentuate the digital health divide for this already disadvantaged population.

To supplement the data on effectiveness of the Vida Sana intervention, this study will provide robust evidence of the potential for implementation and dissemination according to the RE-AIM model. This will result in essential contextual information that health care and public health stakeholders can use to guide implementation decisions for their particular setting. Confirmation of our primary hypothesis and supportive secondary data can critically inform national DPP dissemination and implementation efforts to control obesity and prevent diabetes among high-risk Latino adults in primary care settings.

Acknowledgments

The project described is supported by Award Number R01HS022702 from the Agency for Healthcare Research and Quality and internal funding from the Palo Alto Medical Foundation Research Institute. The content is solely the responsibility of the authors and does not necessarily represent the official views of the Agency for Healthcare Research and Quality or the National Institutes of Health. No sponsor or funding source has a role in the design or conduct of the study; collection, management, analysis or interpretation of the data; or preparation, review or approval of the manuscript.

The authors extend special thanks to Vida Sana participants and their families who make this study possible. The authors also thank the study physician advisors (Meg Durbin, MD, Ed Yu, MD, Patricia Santana, MD, and Sarah Torres, MD), the study Data and Safety Monitoring Board members (William L. Haskell, PhD, Manisha Desai, PhD, Armando Valdez, PhD, and Jeanette

Aviles, MD), and the study team members who have made substantial contributions to the conduct of the study (Veronica Luna, BS, Andrea Blonstein, MBA, RD, Elizabeth Jameiro, MD, Nancy Wittels, MS).

Table 1. Inclusion and exclusion criteria

Inclusion criteria:

- Age(as of date of enrollment):
    - Lower age limit: 18 years
    - Upper age limit: NONE (only exclude for cause, e.g. disease and functional limitations, as detailed below)
- Race/ethnicity: Latino of any race
- Gender: men and women
- Body mass index: ≥24 kg/m^2 (≥22 kg/m^2 if of Asian descent)
- Having pre-diabetes, metabolic syndrome, or both based on the following criteria:
    - Pre-diabetes according to any one of the following criteria:
        - Fasting plasma glucose of 100 to 125 mg/dL or HbA1c of 5.7 to 6.4 if detected by a recent (within the past year), documented, blood-based diagnostic test or by a fasting blood test during study screening
        - Plasma glucose measured 2 hours after a 75 gm glucose load of 140 to 199 mg/dl if detected by a recent (within the past year), documented, blood-based diagnostic test (Oral glucose tolerance test will not be performed for study screening considering participant burden)
        - Clinically diagnosed gestational diabetes mellitus during a previous pregnancy (may be self-reported)
    - Metabolic syndrome according to 3 or more of the following:
        - Waist circumference ≥40 inches in men and ≥35 inches in women (≥35 inches in men and ≥31 inches in women, if of Asian descent)
        - Triglycerides ≥150 mg/dL
        - High-density lipoprotein cholesterol (HDL-C) <40 mg/dL in men and <50 mg/dL in women
        - Systolic blood pressure ≥130 mmHg or diastolic blood pressure ≥85 mmHg
        - Fasting plasma glucose of 100 to 125 mg/dL
- PCP approval of patient contact for study screening
- Able and willing to enroll and provide informed consent, i.e., to meet the time and data collection requirements of the study, be randomized to one of two study arms, participate in follow-up for 24 months, and authorize extraction of relevant information from the EHR
- PAMF patient for ≥1 year and seen in primary care at least once in the preceding 24 months

Exclusion criteria:

- Medical exclusions:
    - Previous diagnosis of diabetes (other than during pregnancy) or diabetes diagnosed as a result of fasting blood glucose or hemoglobin A1c levels obtained through study screening
    - Diagnosis of cancer (other than non-melanoma skin cancer) that is/was active or treated with radiation or chemotherapy within the past 2 years
    - Inability to walk without the assistance of another person
    - Severe medical co-morbidities that require aggressive treatment (e.g., stage 4 or greater renal disease, class III or greater heart failure, unstable coronary artery disease, liver or renal failure)
    - Diagnosis of a terminal illness and/or in hospice care
    - Diagnosis of bipolar disorder or psychotic disorder within the last 2 years, or currently

taking a mood stabilizer or antipsychotic medication

  - Initiation or change in type or dosing of antidepressant medications within 2 months prior to enrollment (The patient will be re-contacted for a later cohort once his/her regimen has been stable for at least 2 months unless the person declines to participate altogether.)
  - Have had or plan to undergo bariatric surgery during the study period
- Other exclusions:
  - Inability to speak, read or understand Spanish or English
  - Having no reliable telephone service
  - Having no regular Internet access via a computer and/or mobile device (e.g., smartphone)
  - Currently pregnant or lactating or planning to become pregnant during the study period
  - Plan to move out of the area during the study period
  - Family/household member of another study participant or of a study staff member
  - Investigator discretion for clinical safety or protocol adherence reasons

Table 2. List of measures and data collection schedule.

Measures	Instrument	Source	BV	12	24
**Primary Outcome**					
Weight	Scale	Biophysical	x	x	x
**Secondary Outcome**					
BMI (height)	Stadiometer	Biophysical	x		
Cardiometabolic risk factors	Blood pressure, waist circumference	Biophysical	x	x	x
Cardiometabolic risk factors	Fasting lipids, fasting glucose and Hemoglobin A1c, insulin, hsCRP	EHR	24 mos pre & post		
Dietary intake	Nutrition Data System for Research 24-hr diet recalls	Interview	x	x	x
Physical activity	7-day Physical Activity Recall	Interview	x	x	x
Health related quality of life	EuroQOL-5D-5L	Self-administered	x	x	x
Obesity-specific quality of life	Obesity-related Problem Scale	Self-administered	x	x	x
Depressive symptoms	Patient Health Questionnaire-9	Self-administered	x	x	x
Sleep habits and quality	PROMIS Sleep disturbance and Sleep Impairment short forms	Self-administered	x	x	x
**Potential effect modifiers**					
Sociodemographics	Age, sex, education, employment, occupation, marital status, household size, income, country of origin		x		
Food security	Household Food Security Scale	Self-administered	x	x	x
Acculturation	Short Acculturation Scale for Hispanics	Self-administered	x		
Health literacy	Short Assessment of Health Literacy–Spanish and English	Interview	x		
**Potential effect mediators**					
Self-efficacy	Weight Efficacy Life-Style Questionnaire; Self-Efficacy for Dietary Change and Exercise	Self-administered	x	x	x
Social support	Social Support for Diet Change & Exercise	Self-administered	x	x	x

Intervention adherence	Group session attendance, number of self-monitoring records and online messages	Intervention process measures	x

Table 3. Summary of quantitative and qualitative measures for the process evaluation

RE-AIM Domains	Example Questions, Data Sources, and Methods<br>Quantitative	Qualitative
Reach of the intended target population	Using patient survey and recruitment tracking data we will assess the percentage and characteristics of participants compared with non-participants.	Via interviews with study staff at the end of the recruitment period we will ask: What were the barriers to and enablers of recruiting participants? Was there variability in these factors related to demographics or other characteristics? How were the barriers addressed? Were the solutions successful? What could be improved to maximize reach?
Adoption by target staff or settings	Using administrative data we will describe the characteristics of participating clinics, and the percentage and characteristics of PCPs who participated.	Via interviews with PCPs and clinic leadership we will ask: What were the barriers to and enablers of clinic and provider participation in patient referrals? Why did the barriers exist? What recommendations do they have for reducing barriers and maximizing adoption?
Implementation success during intervention delivery (***staff perspective***)	Via surveys of lifestyle coach we will assess their perceptions of (1) consistency of intervention procedures, (2) intervention suitability for primary care, and (3) experience with the strategies facilitating intervention delivery (e.g., training, supervision, audit and feedback). We will measure the costs of intervention personnel and supplies.	Via interviews with lifestyle coach, PCPs, and clinic leadership we will ask: What were the barriers to and enablers of delivering the intervention? How might these factors translate (or not) to implementation after the study ends? Were certain components more challenging to deliver than others? What modifications could be made to maximize implementation success?
Implementation success during intervention delivery (***patient perspective***)	We will assess intervention participants' engagement and adherence by monitoring the number of group sessions attended, reasons for missed sessions, secure e-messaging and self-monitoring frequency, and adherence across participant subgroups.	Via interviews with a random sample of participants we will ask: How culturally relevant and acceptable were the knowledge and skills gained? How often did they practice the intervention strategies? What were the perceived benefits? What problems did they encounter? How satisfied were they with program format, materials, and coach performance?
Maintenance of intervention effects in individuals and settings over time	Aim 1 focuses on individual-level sustainability of the intervention effects through 24 months. Additionally, we will assess attrition and adverse events by participant characteristics and treatment condition.	Via interviews with lifestyle coach, PCPs, and clinic leadership we will ask: How could the intervention be integrated into regular care and sustained after the study ends? What resources, policies, and care process redesigns would be needed to maximize sustainability?

Appendix A. Vida Sana Data and Safety Monitoring Plan

The following procedures will be followed to ensure the safety of study participants and the validity and integrity of data in compliance with NIH requirements.

Functions of the Data and Safety Monitoring Board (DSMB). A DSMB in the context of this investigator-initiated randomized controlled trial exists for the purpose of providing the investigators, the cognizant IRB, and the sponsor with objective scientific monitoring of the conduct of the study from the standpoint of ensuring the protection and safety of human subjects and the validity and integrity of the trial. The DSMB will be an independent, advisory body to the investigators and funding agency. To fulfill its functions, the DSMB will review the original protocol and any subsequent amendments, perform expedited monitoring of all serious adverse events (SAEs), perform ongoing monitoring of drop-outs and non-SAEs, determine whether study procedures should be changed or the study should be halted because of serious safety concerns and/or major problems with the study conduct, and perform periodic review of the completeness and validity of data to be used for analysis of safety and efficacy. The DSMB also will monitor implementation of procedures to ensure research participant privacy and data confidentiality.

As in any clinical trial, it is not possible to anticipate all possible adverse events (AEs). We will conduct extensive training with our staff on ascertaining, monitoring, and documenting AEs, serious or not. The study investigators have extensive experience in clinical trials organization and management, including data and safety monitoring for single site and multi-site trials. We have established procedures for rendering first aid in life threatening emergencies.

Membership of the DSMB. The DSMB will consist of 3-5 outside members (not part of the investigative team) with expertise in a variety of disciplines including biobehavioral medicine, preventive medicine, nutrition, physical activity, biostatistics, clinical trial designs, and bioethics of research conduct. In the event of an award, we will work with the AHRQ-designated PO to appoint an appropriate DSMB. The expertise of the members will include the disciplines and skills needed to initially review the protocol and then to monitor trial progress, data quality, and participant safety. The voting members must have no personal stake in the scientific outcomes of the study. They will not be included as authors of publications resulting from the study but will be acknowledged for their contribution. The PI and Reporting Investigator (Dr. Ma) will be responsible for overseeing the preparation of AEs and SAEs and all statistical reports to the DSMB.

Functional Organization of the DSMB. One individual will serve as Chairperson of the DSMB and will communicate by e-mail and telephone conference with the other members on an as-needed basis. Communication pertaining to review of SAEs will occur within a week of receiving any new SAE report. Reporting and communication about other matters will occur on a regular, quarterly basis, for the duration of the study.

DSMB Meetings and Recommendations. The DSMB will convene quarterly, in person or by conference call, with the investigators to review summaries of patient accrual, data collection, the timeliness of data transfer to analysis files, group balance and data concerning the execution of the randomization process, analysis plans and results, and the numbers and characteristics of any SAEs, and the numbers and rates of non-SAEs. At the end of each meeting, DSMB members will make a recommendation regarding the continuation of the trial and the date and format of the next meeting. In addition, there will be an evaluative statement regarding SAEs, protocol exceptions, and other matters of data quality, integrity of the trial, and timeliness. The DSMB's findings and recommendations will be documented in the meeting minutes and transmitted to the Investigators and sponsor for their information and action. A draft of the meeting minutes will be made available to the DSMB Chair for approval prior to distribution of a final version to other DSMB members, the funding agency, and the Investigators.

Monitoring of Safety Data by the DSMB.

Blinded Reporting – Safety information for this study will be reported to the DSMB by group but with the true identity of the treatment groups masked. This will maintain blinding of the investigators, staff responsible for follow-up assessment and data analysis, and the DSMB until the trial is completed. However, if there are extraordinary concerns regarding participant safety during the course of the study, the DSMB may request unblinded data, e.g., on unanticipated SAEs, in order to determine the nature and extent of adverse consequences of the interventions. When this occurs, the unblinded results will not be released to the investigators unless warranted for safety protection of the research participants.

No formal interim analyses are proposed of study outcomes by treatment group. Follow-up data will be reported for all participants, irrespective of treatment assignment, during the course of the study. For purposes of study monitoring, including review of planned outcome analyses, the DSMB may wish to review results with permuted treatment group (i.e., treatment arm randomly assigned) to test the analysis programs. This will maintain blinding of the investigators, staff, and DSMB.

SAEs – Expedited review will occur for all events meeting the NIH definition of SAEs – i.e., any fatal event, immediately life-threatening event, permanently or substantially disabling event, event requiring or prolonging inpatient hospitalization, or congenital anomaly. This also includes any event that study investigators or the DSMB judges to impose a significant hazard, contraindication, side effect, or precaution. *For purposes of this study, all SAEs will be required to be reported to the DSMB, regardless of the study relatedness.* All relevant information will be reported to the DSMB for each SAE including information about the event and its outcome, dosing history of a suspect medication/treatment, concomitant medications, the subject's medical history and current conditions, and all relevant study data. Notification by e-mail and FAX transmittal of all related study forms shall be made to the DSMB within two days of discovery of any unanticipated SAE. Information will be reviewed and a determination made of whether there was any possible relevance to the study.

Non-SAEs – At periodic intervals, the DSMB will be provided with summaries of the numbers and rates of AEs by blinded treatment group. By blinded group is meant an arbitrary labeling (e.g., A, B) that does not reveal the true identity of the groups. These reports will include types of events, severity, and treatment phase. Data on individual non-SAEs is not expected to be needed for this review. At the discretion of the DSMB, however, the Chair may request unblinded and/or individual-level results in order to determine the nature and extent of adverse consequences of the interventions.

Other Safety-Related Reports – It is considered necessary for the purpose of monitoring the safety of the study that the DSMB review not only AEs and SAEs, but other data that may reflect differences in safety between treatment groups. These include treatment retention rates and reasons for dropouts. In addition, changes in BMI and cardiovascular disease risk factors from baseline to follow-up will be reported for all participants, irrespective of treatment assignment, because as noted above, interim outcome analyses by group are not planned in this trial.

Study Stopping Rules – Formal stopping rules for safety, efficacy, and futility are not proposed as part of this application but may be established per recommendations of the DSMB following the funding of the grant. If at any time during the course of the study the DSMB judges that risk to subjects may significantly outweighs the potential benefit, the DSMB shall have the discretion and responsibility to request all necessary information for detailed analyses and, if

warranted, recommend that the study be terminated. Stopping rules for the trial may include stopping because of a significant number of injuries or illnesses that can reasonably be attributed to participation in the study, inability to recruit and measure the required number of participants to conduct the primary outcome analyses, poor intervention quality and delivery, serious deviation from study protocols, or other circumstances that would render the study unlikely to produce scientifically valid findings. The DSMB will carefully weigh the risk of completing the trial as planned against the risk of prematurely stopping the trial for safety or futility.

Monitoring of Data Quality by the DSMB. At least on a quarterly basis during the course of the study, the DSMB will receive a report on data quality and completeness. At a minimum, this will include the following: (1) patient accrual and follow-up completion/retention in relation to goals and timeline; (2) the randomization process and group comparability on the balancing variables; (3) key baseline characteristics of the sample, by blinded group, related to the primary and secondary outcome variables and proposed effect moderators and mediators; (4) indices of intervention adherence; and (5) protocol violations.

Annual DSMB Report to the sponsor. Annually during the course of the study, the DSMB will prepare a summary report of its findings regarding safety and quality based on data received to that point in the study. This report will include a summary of all safety findings, as well as an assessment of protocol compliance and data quality. Any recommendations to improve patient safety, protocol adherence, or data quality will be made in the annual DSMB report. A copy of the annual DSMB report will be sent to the sponsor and the local IRBs along with the annual progress/renewal report.

Requirements for AE Reporting. The PAMFRI IRB requires reporting within 24 hours of any death or unanticipated SAE related to the study, within three days of any emergencies requiring protocol deviation in order to eliminate any suspected immediate hazards to subjects, and within five days of any unanticipated problems involving risk to subjects. This timeline satisfies the requirements of the NIH and those of the IRBs of the consortium institutions involved in this study. An annual report will be submitted to the IRBs of PAMFRI, RTI, and the University of Pittsburgh and to the sponsor summarizing all AEs, serious or not.

References

1. U.S. Census Bureau. Annual Estimates of the Resident Population by Sex, Age, Race, and Hispanic Origin for the United States and States: April 1, 2010 to July 1, 2014. 2015; http://factfinder.census.gov/faces/tableservices/jsf/pages/productview.xhtml?pid=PEP_2013_PEPASR6H&prodType=table. Accessed July 31, 2015.
2. Ogden CL, Carroll MD, Kit BK, Flegal KM. Prevalence of childhood and adult obesity in the United States, 2011-2012. *JAMA.* 2014;311(8):806-814.
3. Flegal KM, Carroll MD, Kit BK, Ogden CL. Prevalence of obesity and trends in the distribution of body mass index among US adults, 1999-2010. *JAMA.* 2012;307(5):491-497.
4. Ogden CL, Carroll MD, Kit BK, Flegal KM. Prevalence of obesity and trends in body mass index among US children and adolescents, 1999-2010. *JAMA.* 2012;307(5):483-490.
5. Mozumdar A, Liguori G. Persistent increase of prevalence of metabolic syndrome among U.S. adults: NHANES III to NHANES 1999-2006. *Diabetes Care.* 2011;34(1):216-219.
6. Roger VL, Go AS, Lloyd-Jones DM, et al. Heart disease and stroke statistics--2012 update: a report from the American Heart Association. *Circulation.* 2012;125(1):e2-e220.
7. Daviglus ML, Talavera GA, Aviles-Santa ML, et al. Prevalence of major cardiovascular risk factors and cardiovascular diseases among Hispanic/Latino individuals of diverse backgrounds in the United States. *JAMA.* 2012;308(17):1775-1784.
8. Wadden TA, Butryn ML, Hong PS, Tsai AG. Behavioral treatment of obesity in patients encountered in primary care settings: a systematic review. *Jama.* 2014;312(17):1779-1791.
9. Aguiar EJ, Morgan PJ, Collins CE, Plotnikoff RC, Callister R. Efficacy of interventions that include diet, aerobic and resistance training components for type 2 diabetes prevention: a systematic review with meta-analysis. *Int J Behav Nutr Phys Act.* 2014;11:2.
10. Ali MK, Echouffo-Tcheugui J, Williamson DF. How effective were lifestyle interventions in real-world settings that were modeled on the diabetes prevention program? *Health Aff (Millwood).* 2012;31(1):67-75.
11. Whittemore R. A systematic review of the translational research on the Diabetes Prevention Program. *Translational behavioral medicine.* 2011;1(3):480-491.
12. Knowler WC, Barrett-Connor E, Fowler SE, et al. Reduction in the incidence of type 2 diabetes with lifestyle intervention or metformin. *N Engl J Med.* 2002;346(6):393-403.
13. Knowler WC, Fowler SE, Hamman RF, et al. 10-year follow-up of diabetes incidence and weight loss in the Diabetes Prevention Program Outcomes Study. *Lancet.* 2009;374(9702):1677-1686.
14. Kramer MK, Kriska AM, Venditti EM, et al. Translating the Diabetes Prevention Program: a comprehensive model for prevention training and program delivery. *Am J Prev Med.* 2009;37(6):505-511.
15. Kramer MK, Kriska AM, Venditti EM, et al. A novel approach to diabetes prevention: evaluation of the Group Lifestyle Balance program delivered via DVD. *Diabetes Res Clin Pract.* 2010;90(3):e60-63.
16. Diabetes Prevention Support Center. Group Lifestyle Balance Materials. *A Modification of the Diabetes Prevention Program's Lifestyle Change Program*. Pittsburgh, PA: University of Pittsburgh; 2011: http://www.diabetesprevention.pitt.edu/index.php/for-the-public/group-lifestyle-balance-materials/. Accessed October 19, 2015.
17. Centers for Disease Control and Prevention (CDC). *Centers for Disease Control and Prevention Diabetes Prevention Recognition Program Standards and Operating Procedures.* Atlanta, Georgia: Centers for Disease Control and Prevention;2011.

18. Greenwood DA, Kramer MK, Hankins AI, Parise CA, Fox A, Buss KA. Adapting the Group Lifestyle Balance Program for Weight Management Within a Large Health Care System Diabetes Education Program. *The Diabetes educator.* 2014;40(3):299-307.
19. Kramer MK, Miller R, Venditti E, Orchard TO. Group lifestyle intervention for diabetes prevention in those with metabolic syndrome in primary care practice. *Diabetes Care.* 2006;55((suppl)):A517.
20. McTigue KM, Conroy MB, Bigi L, Murphy C, McNeil M. Weight loss through living well: translating an effective lifestyle intervention into clinical practice. *Diabetes Educ.* 2009;35(2):199-204, 208.
21. Kramer MK, McWilliams JR, Chen HY, Siminerio LM. A community-based diabetes prevention program: evaluation of the group lifestyle balance program delivered by diabetes educators. *Diabetes Educ.* 2011;37(5):659-668.
22. Kramer MK, Molenaar DM, Arena VC, et al. Improving employee health: evaluation of a worksite lifestyle change program to decrease risk factors for diabetes and cardiovascular disease. *Journal of occupational and environmental medicine / American College of Occupational and Environmental Medicine.* 2015;57(3):284-291.
23. Sevick MA, Zickmund S, Korytkowski M, et al. Design, feasibility, and acceptability of an intervention using personal digital assistant-based self-monitoring in managing type 2 diabetes. *Contemp Clin Trials.* 2008;29(3):396-409.
24. Burke LE, Conroy MB, Sereika SM, et al. The effect of electronic self-monitoring on weight loss and dietary intake: a randomized behavioral weight loss trial. *Obesity (Silver Spring).* 2011;19(2):338-344.
25. Neuhausera L, Kreps GL. eHealth communication and behavior change: promise and performance. *Soc Semiot.* 2010;20(1):9-27.
26. Krukowski RA, Harvey-Berino J, West DS. Obesity. In: Cucciare MA, Weingardt KR, eds. *Using technology to support evidence-based behavioral health practices: a clinician's guide*. New York: Routledge; 2009.
27. Krebs P, Prochaska JO, Rossi JS. A meta-analysis of computer-tailored interventions for health behavior change. *Prev Med.* 2010;51(3-4):214-221.
28. Neve M, Morgan PJ, Jones PR, Collins CE. Effectiveness of web-based interventions in achieving weight loss and weight loss maintenance in overweight and obese adults: a systematic review with meta-analysis. *Obes Rev.* 2010;11(4):306-321.
29. Arem H, Irwin M. A review of web-based weight loss interventions in adults. *Obes Rev.* 2011;12(5):e236-243.
30. Manzoni GM, Pagnini F, Corti S, Molinari E, Castelnuovo G. Internet-based behavioral interventions for obesity: an updated systematic review. *Clinical practice and epidemiology in mental health : CP & EMH.* 2011;7:19-28.
31. Spring B, Duncan JM, Janke EA, et al. Integrating technology into standard weight loss treatment: a randomized controlled trial. *JAMA Intern Med.* 2013;173(2):105-111.
32. Burke LE, Ma J, Azar KM, et al. Current Science on Consumer Use of Mobile Health for Cardiovascular Disease Prevention: A Scientific Statement From the American Heart Association. *Circulation.* 2015;132(12):1157-1213.
33. Ma J, King AC, Wilson SR, Xiao L, Stafford RS. Evaluation of lifestyle interventions to treat elevated cardiometabolic risk in primary care (E-LITE): a randomized controlled trial. *BMC Fam Pract.* 2009;10:71.
34. Yank V, Stafford RS, Rosas LG, Ma J. Baseline reach and adoption characteristics in a randomized controlled trial of two weight loss interventions translated into primary care: A structured report of real-world applicability. *Contemp Clin Trials.* 2013;34(1):126-135.

35. Ma J, Xiao L, Blonstein A. Measurement of self-monitoring web technology acceptance and use in an eHealth weight-loss trial. *Telemed & eHealth.* 2013 (in press).
36. Blonstein AC, Yank V, Stafford RS, Wilson SR, Rosas LG, Ma J. Translating an evidence-based lifestyle intervention program into primary care: lessons learned. *Health Promot Pract.* 2013;14(4):491-497.
37. Ma J, Yank V, Xiao L, et al. Translating the Diabetes Prevention Program lifestyle intervention for weight loss into primary care: a randomized trial. *JAMA Intern Med.* 2013;173(2):113-121.
38. Xiao L, Yank V, Wilson SR, Lavori PW, Ma J. Two-year weight-loss maintenance in primary care-based Diabetes Prevention Program lifestyle interventions. *Nutrition & diabetes.* 2013;3:e76.
39. Appel LJ, Clark JM, Yeh HC, et al. Comparative effectiveness of weight-loss interventions in clinical practice. *N Engl J Med.* 2011;365(21):1959-1968.
40. Wadden TA, Volger S, Sarwer DB, et al. A two-year randomized trial of obesity treatment in primary care practice. *N Engl J Med.* 2011;365(21):1969-1979.
41. Riley WT, Rivera DE, Atienza AA, Nilsen W, Allison SM, Mermelstein R. Health behavior models in the age of mobile interventions: are our theories up to the task? *Translational behavioral medicine.* 2011;1(1):53-71.
42. Rosas LG, Lv N, Azar K, Xiao L, Yank V, Ma J. Applying the Pragmatic-Explanatory Continuum Indicator Summary Model in a Primary Care-Based Lifestyle Intervention Trial. *Am J Prev Med.* 2015;49(3 Suppl 2):S208-214.
43. Sommers BD, Gunja MZ, Finegold K, Musco T. Changes in Self-reported Insurance Coverage, Access to Care, and Health Under the Affordable Care Act. *JAMA.* 2015;314(4):366-374.
44. Ferguson WJ, Candib LM. Culture, language, and the doctor-patient relationship. *FMCH Publications and Presentations.* 2002:61.
45. Flores G. Culture and the patient-physician relationship: achieving cultural competency in health care. *The Journal of pediatrics.* 2000;136(1):14-23.
46. Control CfD, Prevention. Building our understanding: Culture insights. Communicating with Hispanic/Latinos. 2013.
47. Glasgow RE, McKay HG, Piette JD, Reynolds KD. The RE-AIM framework for evaluating interventions: what can it tell us about approaches to chronic illness management? *Patient Educ Couns.* 2001;44(2):119-127.
48. Poston L, Harthoorn LF, Van Der Beek EM. Obesity in pregnancy: implications for the mother and lifelong health of the child. A consensus statement. *Pediatric research.* 2011;69(2):175-180.
49. Grundy SM, Cleeman JI, Daniels SR, et al. Diagnosis and management of the metabolic syndrome: an American Heart Association/National Heart, Lung, and Blood Institute Scientific Statement. *Circulation.* 2005;112(17):2735-2752.
50. NIH and National Heart Lung Blood Institute. *Clinical guidelines on the identification, evaluation, and treatment of overweight and obesity in adults: the evidence report.* Rockville, MD: DHHS, Public Health Service; October 1998. NIH Publication No. 00-4084.
51. Pickering TG, Hall JE, Appel LJ, et al. Recommendations for blood pressure measurement in humans and experimental animals: Part 1: blood pressure measurement in humans: a statement for professionals from the Subcommittee of Professional and Public Education of the American Heart Association Council on High Blood Pressure Research. *Hypertension.* 2005;45(1):142-161.
52. Measures from the PhenX Toolkit version February 4 2011, Ver 4.2 (www.phenxtoolkit.org). PhenX (consensus measures of Phenotypes and eXposures) is supported by NHGRI award No. U01 HG004597. Accessed January 15, 2015.
53. Xiao L, Lavori PW, Wilson SR, Ma J. Comparison of dynamic block randomization and minimization in randomized trials: a simulation study. *Clin Trials.* 2011;8(1):59-69.

54. Ma J, Strub P, Xiao L, et al. Behavioral weight loss and physical activity intervention in obese adults with asthma. A randomized trial. *Ann Am Thorac Soc.* 2015;12(1):1-11.
55. Ma J, Strub P, Camargo CA, Jr., et al. The Breathe Easier through Weight Loss Lifestyle (BE WELL) Intervention: a randomized controlled trial. *BMC Pulm Med.* 2010;10:16.
56. Marín G. Issues in the measurement of acculturation among Hispanics. In: Geisinger KF, ed. *Psychological testing of Hispanics* Washington, DC: American Psychological Association; 1992:235-251.
57. Marin G, Gamba RJ. A new measurement of acculturation for Hispanics: the Bidimensional Acculturation Scale for Hispanics (BAS). *Hisp J Behav Sci.* 1996;18:297-316.
58. Prochaska JO, DiClemente CC. Stages and processes of self-change of smoking: toward an integrative model of change. *J Consult Clin Psychol.* 1983;51(3):390-395.
59. Bandura A. *Social Foundations of Thought and Action: A Social Cognitive Theory.* Englewood Cliffs, N.J.: Prentice Hall; 1986.
60. Winett RA, Tate DF, Anderson ES, Wojcik JR, Winett SG. Long-term weight gain prevention: a theoretically based Internet approach. *Prev Med.* 2005;41(2):629-641.
61. U.S. Department of Agriculture. MyPlate. http://www.choosemyplate.gov/MyPlate. Accessed March 8, 2016.
62. Burke LE, Wang J, Sevick MA. Self-monitoring in weight loss: a systematic review of the literature. *J Am Diet Assoc.* 2011;111(1):92-102.
63. Perrin A, Duggan M. Americans' internet access: 2000–2015. *Pew Research Center, Washington.* 2015.
64. Bellg AJ, Borrelli B, Resnick B, et al. Enhancing treatment fidelity in health behavior change studies: best practices and recommendations from the NIH Behavior Change Consortium. *Health Psychol.* 2004;23(5):443-451.
65. Ma J, Strub P, Lavori PW, et al. DASH for asthma: A pilot study of the DASH diet in not-well-controlled adult asthma. *Contemp Clin Trials.* 2013;35(2):55-67.
66. Ma J, Yank V, Lv N, et al. Research aimed at improving both mood and weight (RAINBOW) in primary care: A type 1 hybrid design randomized controlled trial. *Contemp Clin Trials.* 2015;43:260-278.
67. Ma J, Strub P, Lv N, et al. Pilot randomized trial of a healthy eating behavioural intervention in uncontrolled asthma. *Eur Respir J.* 2015 (accepted for publication).
68. Rosas LG, Thiyagarajan S, Goldstein BA, et al. The effectiveness of two community-based weight loss strategies among obese, low-income US Latinos. *Journal of the Academy of Nutrition and Dietetics.* 2015;115(4):537-550 e532.
69. Ma J, Berra K, Haskell WL, et al. Case management to reduce risk of cardiovascular disease in a county health care system. *Arch Intern Med.* 2009;169(21):1988-1995.
70. Moshfegh AJ, Goldman J, Lacomb R, Perloff B, Cleveland L. Research results using the new USDA Automated Multiple-Pass Method. *FASEB J.* 2001;15(4):A278 (abstr).
71. Conway JM, Ingwersen LA, Vinyard BT, Moshfegh AJ. Effectiveness of the US Department of Agriculture 5-step multiple-pass method in assessing food intake in obese and nonobese women. *Am J Clin Nutr.* 2003;77(5):1171-1178.
72. Conway JM, Ingwersen LA, Moshfegh AJ. Accuracy of dietary recall using the USDA five-step multiple-pass method in men: an observational validation study. *J Am Diet Assoc.* 2004;104(4):595-603.
73. Blair SN, Haskell WL, Ho P, et al. Assessment of habitual physical activity by a seven-day recall in a community survey and controlled experiments. *Am J Epidemiol.* 1985;122(5):794-804.
74. Badia X, Schiaffino A, Alonso J, Herdman M. Using the EuroQol 5-D in the Catalan general population: feasibility and construct validity. *Qual Life Res.* 1998;7(4):311-322.

75. Karlsson J, Taft C, Sjostrom L, Torgerson JS, Sullivan M. Psychosocial functioning in the obese before and after weight reduction: construct validity and responsiveness of the Obesity-related Problems scale. *Int J Obes Relat Metab Disord.* 2003;27(5):617-630.
76. Spitzer RL, Kroenke K, Williams JB. Validation and utility of a self-report version of PRIME-MD: the PHQ primary care study. Primary Care Evaluation of Mental Disorders. Patient Health Questionnaire. *JAMA.* 1999;282(18):1737-1744.
77. Kroenke K, Spitzer RL, Williams JB. The PHQ-9: validity of a brief depression severity measure. *J Gen Intern Med.* 2001;16(9):606-613.
78. Yu L, Buysse DJ, Germain A, et al. Development of short forms from the PROMIS sleep disturbance and Sleep-Related Impairment item banks. *Behavioral sleep medicine.* 2011;10(1):6-24.
79. United States Department of Agriculture. U.S. Household Food Security Survey Module. 2015; http://www.ers.usda.gov/topics/food-nutrition-assistance/food-security-in-the-us/survey-tools.aspx#Spanish. Accessed September 23, 2015.
80. Marín G, Sabogal F, VanOss Marín B, Otero-Sabogal F, Pérez-Stable EJ. Development of a short acculturation scale for Hispanics. *Hispanic Journal of Behavioral Sciences.* 1987;9:183-205.
81. Lee SY, Stucky BD, Lee JY, Rozier RG, Bender DE. Short Assessment of Health Literacy-Spanish and English: a comparable test of health literacy for Spanish and English speakers. *Health Serv Res.* 2010;45(4):1105-1120.
82. Ruiz VM, Berrocal C, López AE, Rivas T. Factor Analysis of the Spanish Version of the Weight Efficacy Life-Style Questionnaire *Educational and Psychological Measurement.* 2002;62(3):539-555
83. Sallis JF, Pinski RB, Grossman RM, Patterson TL, Nader PR. The development of self-efficacy scales for health-related diet and exercise behaviors. *Heath Educ Res.* 1988;3:283-292.
84. Sallis JF, Grossman RM, Pinski RB, Patterson TL, Nader PR. The development of scales to measure social support for diet and exercise behaviors. *Prev Med.* 1987;16(6):825-836.
85. Curran GM, Bauer M, Mittman B, Pyne JM, Stetler C. Effectiveness-implementation hybrid designs: combining elements of clinical effectiveness and implementation research to enhance public health impact. *Med Care.* 2012;50(3):217-226.
86. Glasgow RE, Vogt TM, Boles SM. Evaluating the public health impact of health promotion interventions: the RE-AIM framework. *Am J Public Health.* 1999;89(9):1322-1327.
87. Glasgow RE, Klesges LM, Dzewaltowski DA, Bull SS, Estabrooks P. The future of health behavior change research: what is needed to improve translation of research into health promotion practice? *Ann Behav Med.* 2004;27(1):3-12.
88. Kessler RS, Purcell EP, Glasgow RE, Klesges LM, Benkeser RM, Peek CJ. What does it mean to "employ" the RE-AIM model? *Eval Health Prof.* 2013;36(1):44-66.
89. Ma J, Yank V, Lv N, et al. Research Aimed at Improving Both Mood and Weight (RAINBOW) in Primary Care: A Type 1 hybrid design randomized controlled trial. *Contemporary clinical trials.* 2015.
90. Littell RC, Milliken GA. *SAS System for Mixed Models.* Cary, NC: SAS Institute Inc; 1996.
91. Brown H, Prescott R. *Applied Mixed Models in Medicine.* Chichester, England: John Wiley & Sons, Inc.; 1999.
92. Verbeke G, Molenberghs G. *Linear Mixed Models for Longitudinal Data.* New York: Springer-Verlag; 2000.
93. Shao J, Yu X, Zhong B. A theory for testing hypotheses under covariate-adaptive randomization. *Biometrika.* 2010;97(2):347-360.
94. Lavori PW, Dawson R, Shera D. A multiple imputation strategy for clinical trials with truncation of patient data. *Stat Med.* 1995;14(17):1913-1925.

95. Schafer JL. *Analysis of Incomplete Multivariate Data.* New York: Chapman and Hall; 1997.
96. Tang L, Duan N, Klap R, Asarnow JR, Belin TR. Applying permutation tests with adjustment for covariates and attrition weights to randomized trials of health-services interventions. *Stat Med.* 2009;28(1):65-74.
97. DiCiccio TJ, Efron B. Bootstrap confidence intervals. *Statistic Sci.* 1996;11(3):189-228.
98. MacKinnon DP, Lockwood CM, Hoffman JM, West SG, Sheets V. A comparison of methods to test mediation and other intervening variable effects. *Psychol Methods.* 2002;7(1):83-104.
99. MacKinnon DP, Fritz MS, Williams J, Lockwood CM. Distribution of the product confidence limits for the indirect effect: program PRODCLIN. *Behav Res Methods.* 2007;39(3):384-389.
100. *ATLAS.ti. Version 7.5.* [computer program]. Berlin, Germany: Scientific Software Development; 2014.
101. Elo S, Kyngas H. The qualitative content analysis process. *Journal of advanced nursing.* 2008;62(1):107-115.
102. Patton MQ. *Qualitative research and evaluation methods.* San Francisco: Sage Publications; 2002.
103. Kraemer HC. To increase power in randomized clinical trials without increasing sample size. *Psychopharmacol Bull.* 1991;27(3):217-224.
104. Thomas PR. *Weighing the Options: Criteria for Evaluating Weight-Management Programs.* Washington, DC: Committee to Develop Criteria for Evaluating the Outcomes of Approaches to Prevent and Treat Obesity, Institute of Medicine;1995.
105. Stevens J, Truesdale KP, McClain JE, Cai J. The definition of weight maintenance. *Int J Obes (Lond).* 2006;30(3):391-399.
106. ICH Harmonised Tripartite Guideline. Statistical principles for clinical trials. International Conference on Harmonisation E9 Expert Working Group. *Stat Med.* 1999;18(15):1905-1942.
107. Bender R, Lange S. Adjusting for multiple testing--when and how? *J Clin Epidemiol.* 2001;54(4):343-349.
108. Harris PA, Taylor R, Thielke R, Payne J, Gonzalez N, Conde JG. Research electronic data capture (REDCap)--a metadata-driven methodology and workflow process for providing translational research informatics support. *J Biomed Inform.* 2009;42(2):377-381.
109. Shaibi GQ, Greenwood-Ericksen MB, Chapman CR, Konopken Y, Ertl J. Development, Implementation, and Effects of Community-Based Diabetes Prevention Program for Obese Latino Youth. *J Prim Care Community Health.* 2010;1(3):206-212.
110. Ruggiero L, Oros S, Choi YK. Community-based translation of the diabetes prevention program's lifestyle intervention in an underserved Latino population. *Diabetes Educ.* 2011;37(4):564-572.
111. Merriam PA, Tellez TL, Rosal MC, et al. Methodology of a diabetes prevention translational research project utilizing a community-academic partnership for implementation in an underserved Latino community. *BMC Med Res Methodol.* 2009;9:20.
112. Ockene IS, Tellez TL, Rosal MC, et al. Outcomes of a Latino Community-Based Intervention for the Prevention of Diabetes: The Lawrence Latino Diabetes Prevention Project. *Am J Public Health.* 2012;102(2):336-342.
113. Hall DL, Lattie EG, McCalla JR, Saab PG. Translation of the Diabetes Prevention Program to Ethnic Communities in the United States. *Journal of immigrant and minority health / Center for Minority Public Health.* 2015.
114. Roger VL, Go AS, Lloyd-Jones DM, et al. Heart disease and stroke statistics--2012 update: a report from the American Heart Association. *Circulation.* 2012;125(1):e2-e220.
115. Rosas LG, Thiyagarajan S, Goldstein BA, et al. The Effectiveness of Two Community-Based Weight Loss Strategies among Obese, Low-Income US Latinos. *Journal of the Academy of Nutrition and Dietetics.* 2015.

116. Krukowski RA, West DS, Harvey-Berino J. Recent advances in internet-delivered, evidence-based weight control programs for adults. *Journal of diabetes science and technology.* 2009;3(1):184-189.
117. Spring B, Duncan JM, Janke EA, et al. Integrating Technology Into Standard Weight Loss Treatment: A Randomized Controlled Trial. *Arch Intern Med.* 2012:1-7.
118. Lopez MH, Gonzalez-Barrera A, Patten E. Closing the Digital Divide: Latinos and Technology Adoption. *Religion.* 2014.
119. Sarasohn-Kahn J. *How smartphones are changing health care for consumers and providers.* California HealthCare Foundation;2010.
120. Smith A. U.S. Smartphone Use in 2015. 2015; http://www.pewinternet.org/2015/04/01/us-smartphone-use-in-2015/. Accessed March 2, 2016.
121. Horrigan JB, Duggan M. Home Broadband 2015. 2015; http://www.pewinternet.org/2015/12/21/home-broadband-2015/. Accessed March 8, 2016.
122. Anderson M. Racial and ethnic differences in how people use mobile technology. 2015; http://www.pewresearch.org/fact-tank/2015/04/30/racial-and-ethnic-differences-in-how-people-use-mobile-technology/. Accessed March 8, 2016.
123. King AC, Bickmoreb TW, Camperoc MI, Pruittc LA, Yin JL. Employing Virtual Advisors in Preventive Care for Underserved Communities: Results From the COMPASS Study. *J Health Commun.* 2013.
124. Winter SJ, Goldman Rosas L, Padilla Romero P, et al. Using Citizen Scientists to Gather, Analyze, and Disseminate Information About Neighborhood Features That Affect Active Living. *J Immigr Minor Health.* 2015.
