# Supplementary material for: Effect of a Culturally Adapted Behavioral Intervention for Latino Adults on Weight Loss Over 2 Years: A Randomized Clinical Trial
Source: JAMA Netw Open. 2020 Dec 18;3(12):e2027744. doi: 10.1001/jamanetworkopen.2020.27744 (PMC7749441; doi:10.1001/jamanetworkopen.2020.27744)
Supplement: Supplement 2. — eTable 1. Sensitivity Analysis of Primary Outcome Using Study-Measured Weights eTable 2. Sensitivity Analysis Using Bootstrap Results for the Treatment Effects on Weight Changes From Baseline at 12 and 24 Months [file jamanetwopen-e2027744-s002.pdf]

Supplemental Online Content

Rosas LG, Lv N, Xiao L, et al. Effect of a culturally adapted behavioral intervention for Latino adults on weight loss over 2 years: a randomized clinical trial. *JAMA Netw Open*. 2020;3(12):e2027744. doi:10.1001/jamanetworkopen.2020.27744

eTable 1. Sensitivity Analysis of Primary Outcome Using Study-Measured Weights

eTable 2. Sensitivity Analysis Using Bootstrap Results for the Treatment Effects on Weight Changes From Baseline at 12 and 24 Months

This supplemental material has been provided by the authors to give readers additional information about their work.

© 2020 Rosas LG et al. *JAMA Network Open.*

eTable 1. Sensitivity Analysis of Primary Outcome Using Study-Measured Weights

	**Unadjusted mean change ± SD**		**Adjusted mean treatment difference**	**P value***
**Outcome measures**	**Intervention**	**Usual care**	**(95% CI)***	
**Primary outcome**				
Weight in kg, n†=78, 92				
12 months	-2.8 ± 6.2	-0.3 ± 3.9	-2.5 (-4.1, -0.9)	0.002
24 months	-1.3 ± 6	-1.4 ± 7.4	0.3 (-1.8, 2.4)	0.78

* Adjusted analysis for intervention vs usual care: Adjusted differences in means and 95% CIs were calculated using model-based estimates. Mixed-effects models accounting for the random effects of repeated measures and primary care physicians and adjusted for baseline value of the outcome of interest, study site, age, female, BMI, waist circumference, and short acculturation scale for Hispanics.

†Analysis for the outcome included intervention and usual care participants with follow-up data at 12 and/or 24 months.

eTable 2. Sensitivity Analysis Using Bootstrap Results for the Treatment Effects on Weight Changes From Baseline at 12 and 24 Months

Weight in kg	2.5%	50%	97.5%
12 Months	-3.5	-1.8	-0.2
24 Months	-1.3	0.5	2.5

© 2020 Rosas LG et al. *JAMA Network Open.*
