# Supplementary material for: Effect of a Culturally Adapted Behavioral Intervention for Latino Adults on Weight Loss Over 2 Years: A Randomized Clinical Trial
Source: JAMA Netw Open. 2020 Dec 18;3(12):e2027744. doi: 10.1001/jamanetworkopen.2020.27744 (PMC7749441; doi:10.1001/jamanetworkopen.2020.27744)
Supplement: Supplement 3. — Data Sharing Statement [file jamanetwopen-e2027744-s003.pdf]

Data Sharing Statement

Goldman Rosas. Effect of a Culturally Adapted Behavioral Intervention for Latino Adults on Weight Loss Over 2 Years. *JAMA Netw Open*. Published December 18, 2020. doi:10.1001/jamanetworkopen.2020.27744

Data
Data available: Yes
Data types: Deidentified participant data, Data dictionary
How to access data: Data can be requested by contacting the corresponding author.
When available: With publication

Supporting Documents
Document types: None

Additional Information
Who can access the data: Researcher whose proposed us of the data has been approved.
Types of analyses: Data will be made available to researchers with a specific purpose that is approved by the study team.
Mechanisms of data availability: Data will be made available without support from the study team and only with a signed data use agreement.
